# Longitudinal analysis of memory Tfh cells and antibody response following CoronaVac vaccination

**DOI:** 10.1172/jci.insight.168437

**Published:** 2023-08-08

**Authors:** Pengcheng Zhou, Cheng Cao, Tuo Ji, Ting Zheng, Yaping Dai, Min Liu, Junfeng Jiang, Daoqi Sun, Zhonghu Bai, Xiaojie Lu, Fang Gong

**Affiliations:** 1 Department of Laboratory Medicine, Affiliated Hospital of Jiangnan University, Wuxi, China.; 2 The University of Queensland Diamantina Institute, Brisbane, Queensland, Australia.; 3 Wuxi School of Medicine, Jiangnan University, Wuxi, China.; 4 Department of Central Laboratory, The Second People’s Hospital of Lianyungang City (Cancer Hospital of Lianyungang), Lianyungang, China.; 5 Hospital for Special Surgery, Weill Cornell Medical College, New York, New York, USA.; 6 Department of Laboratory Medicine, The Fifth People’s Hospital of Wuxi, Wuxi, China.; 7 School of Biotechnology, Jiangnan University, Wuxi, China.

**Keywords:** COVID-19, Infectious disease, Adaptive immunity, T cells

## Abstract

The inactivated vaccine CoronaVac is one of the most widely used COVID-19 vaccines globally. However, the longitudinal evolution of the immune response induced by CoronaVac remains elusive compared with other vaccine platforms. Here, we recruited 88 healthy individuals who received 3 doses of CoronaVac vaccine. We longitudinally evaluated their polyclonal and antigen-specific CD4^+^ T cells and neutralizing antibody response after receiving each dose of vaccine for over 300 days. Both the second and third doses of vaccine induced robust spike-specific neutralizing antibodies, with a third vaccine further increasing the overall magnitude of antibody response and neutralization against Omicron sublineages B.1.1.529, BA.2, BA.4/BA.5, and BA.2.75.2. Spike-specific CD4^+^ T cells and circulating T follicular helper (cTfh) cells were markedly increased by the second and third dose of CoronaVac vaccine, accompanied by altered composition of functional cTfh cell subsets with distinct effector and memory potential. Additionally, cTfh cells were positively correlated with neutralizing antibody titers. Our results suggest that CoronaVac vaccine–induced spike-specific T cells are capable of supporting humoral immunity for long-term immune protection.

## Introduction

Since the onset of the COVID-19 pandemic in 2020, SARS-CoV-2 has evolved into several sublineages, including Alpha, Beta, Gamma, Delta, and Omicron (1). Such continuous evolution resulted in rapid and divergent mutations from Omicron, with further Omicron sublineages such as B1.1.529, BA.2, BA.3, BA.4/5, BA.2.75, and BQ1.1 currently spreading in many countries. Despite the significant success of human vaccine efforts in preventing severe disease caused by both ancestral and emerging strains of SARS-CoV-2, there are still significant gaps in our understanding of the mechanisms of vaccine-induced immune protection against emerging variants. These gaps are particularly evident in studies of different vaccine platforms, indicating an inequitable distribution of knowledge. Unlike mRNA vaccines, the evolution and persistence of human immune response elicited by vaccine platforms such as inactivated vaccines or protein vaccines after the second and third booster doses are poorly understood. Recent evidence has shown that a homologous third dose of CoronaVac is associated with an efficient increase in SARS-CoV-2–specific antibodies (2–4). However, the human immune response and antibody neutralizing capacity against ancestral and Omicron sublineages, including BA.2, BA.4/5, and BA.2.75.2, by a third dose of CoronaVac vaccine remain largely unexplored.

In addition to serologic antibodies, antigen-specific CD4^+^ T cells, especially T follicular helper (Tfh) cells, are critical for long-term immune protection (5–7). mRNA vaccines induce robust antigen-specific memory CD4^+^ T cells and Tfh cells (8, 9). However, the dynamics of CoronaVac-induced memory CD4^+^ T cells and their relationship with prolonged antibody response remains poorly understood. It is also crucial to determine whether certain subsets of human Tfh cells exhibit superior effector or memory potential in response to a vaccine longitudinally. Here, we recruited 88 healthy individuals in a longitudinal cohort who received 3 doses of inactivated CoronaVac vaccines. To understand the longevity and nature of human immune response induced by CoronaVac vaccine, we evaluated polyclonal and antigen-specific Tfh cells, neutralizing antibodies, and their relationships following primary, secondary, and third booster doses of CoronaVac vaccinations.

## Results

### Spike-specific antibody response elicited by CoronaVac vaccine over time.

**Figure 1:**
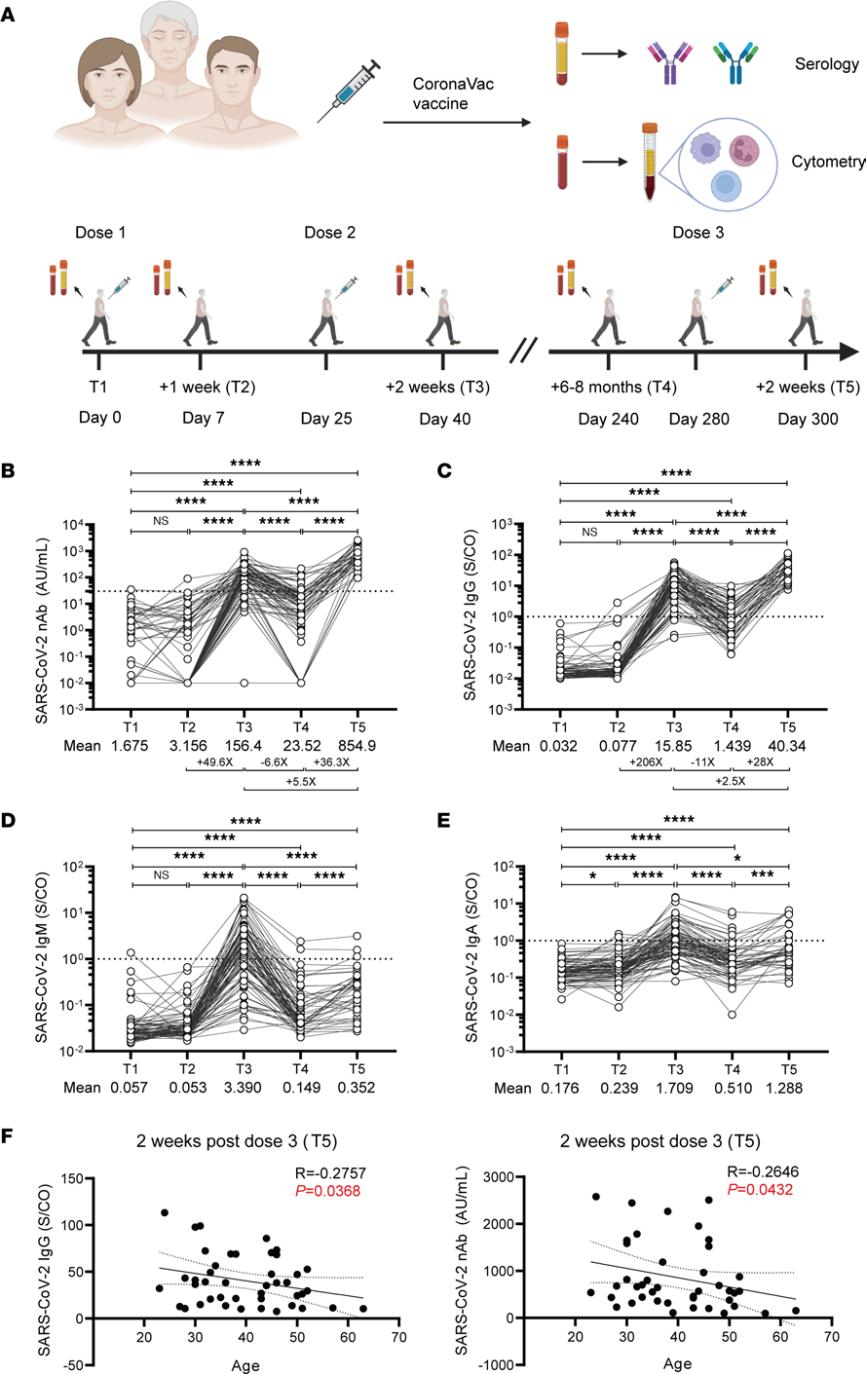
Dynamics of SARS-CoV-2–specific antibody responses. (**A**) Schematics (created with BioRender.com). (**B**) SARS-CoV-2 nAb titer, (**C**) anti-spike IgG titer, (**D**) anti-spike IgM, and (**E**) anti-spike IgA antibodies in vaccinated individuals at 5 time points, including prevaccination (T1), 1 week after the first dose (T2), 2 weeks after the second dose (T3), 6–8 months after the second dose (T4), as well as 2 weeks after the third dose (T5). (**F**) Correlation between age and SARS-CoV-2 IgG and nAb titers at 2 weeks after the third dose (T5). Each dot represents an individual. “+X” indicates fold-changes for selected comparisons. “–X” indicates decreased fold-changes for selected comparisons. The dashed line indicates the cutoff value, and the samples above the dashed line are considered reactive while those below are considered nonreactive. Statistics were calculated using Wilcoxon’s matched pairs signed ranks test for comparison between time points (**B**–**E**). *P < 0.05; **P < 0.01; ***P < 0.001; ****P < 0.0001. The 2-tailed, nonparametric Spearman’s rank correlation was used in **F**. *P* and *R* values were indicated. S/CO, sample RLU/cutoff value.

A total of 88 healthy individuals were recruited in this study, where we longitudinally followed them over 300 days. We collected 390 samples at 5 time points following their 3 doses of vaccination (Figure 1A). The 5 time points are prevaccination baseline (T1), 1 week after dose 1 (T2), 2 weeks after dose 2 (T3), 6–8 months after dose 2 (T4), and 2 weeks after a boost dose 3 (T5). This study design allowed us to monitor immunological alterations, especially the induction, maintenance, waning, and boosting of antigen-specific immune responses to the vaccine in a relatively long period.

At the baseline (T1), all participants had undetectable levels of neutralizing antibodies (nAb). Consistent with previous reports (10–12), the second dose of CoronaVac vaccine substantially enhanced nAb responses against the wild-type (WT) SARS-CoV-2, with the mean value of nAb rising from only 3.156 AU/mL after the primary dose (T2) to 156.4 AU/mL after the second dose (T3). Different from mRNA vaccine–induced antibody response that remains at a relatively high level 6 months after the second dose of vaccine (13–15), the average nAb elicited by CoronaVac vaccine dropped rapidly to 23.52 AU/mL after 6–8 months (T4). Importantly, a third dose of CoronaVac vaccine significantly boosted nAb responses, and the seropositivity rate of nAb rapidly reached 100% (Table 1). Moreover, a third dose of vaccine also significantly enhanced the magnitude of nAb levels (*P* < 0.0001). The average level of nAb reached 854.9 AU/mL at 2 weeks after dose 3 (T5), 5.5-fold higher than that at T3 and 36.3-fold higher than that at T4 (Figure 1B). Similarly, the seropositivity rate and the amount of anti-spike IgG increased significantly after the second vaccine dose, with a continued increase after the third vaccination. The mean value of anti-spike IgG at T5 increased 2.5-fold and 28-fold compared with values at T3 and T4, respectively (Figure 1C). Spike-specific IgM (Figure 1D) and IgA (Figure 1E) displayed a similar kinetics as IgG and nAb during the primary 2-dose vaccine series. By contrast, a third vaccination did not markedly increase IgM and IgA response. The seropositivity rates for anti-spike IgM and anti-spike IgA after the third dose (T5) were much lower than that observed after the second dose (T3) (Table 1). We also compared the effects of vaccination interval during the primary and secondary dose and found minor impacts on the subsequent immune response (Supplemental Figure 1, A and B; supplemental material available online with this article; https://doi.org/10.1172/jci.insight.168437DS1). Notably, we observed that anti-WT SARS-CoV-2 IgG and nAb after the third dose of vaccination were negatively correlated with age (Figure 1F). Together, these results suggest that the second and third CoronaVac vaccine induced robust nAbs, with a third booster further increasing the response.

### Plasma neutralization against variants of concern.

**Figure 2:**
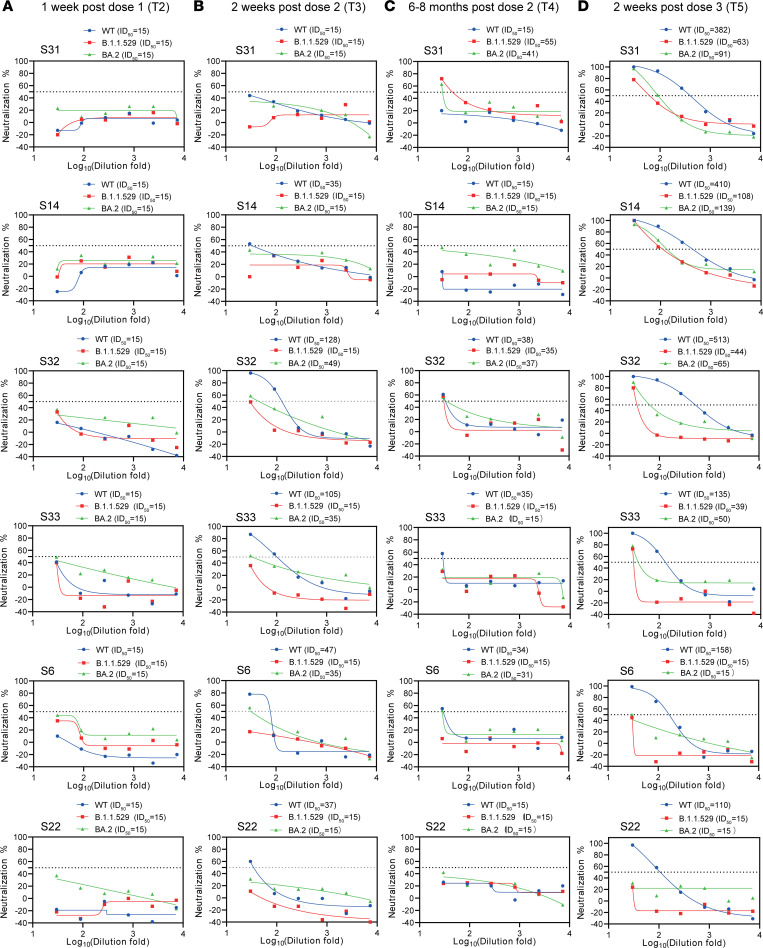
Neutralizing activities against SARS-CoV-2 WT and variants of concern. Pseudovirus neutralization titers against SARS-CoV-2 WT, B.1.1.529, and BA.2 variant using plasma samples from 6 randomly selected individuals. The sera were collected at 1 week after the first dose (T2) (**A**), 2 weeks after the second dose (T3) (**B**), 6–8 months after the second dose (T4) (**C**), and 2 weeks after the third dose (T5) (**D**), respectively. Patient numbers and ID_50_ of different variants are shown at the top of each graph. The horizontal dashed lines indicate 50% of the pseudovirus neutralization (ID_50_).

To examine the neutralizing efficacy and breadth of antibodies elicited by CoronaVac vaccine, especially their evolution and persistence following each vaccination, pseudovirus neutralization was applied, and 50% inhibitory dose (ID_50_) was used to calculate the plasma neutralization against ancestral SARS-CoV-2 (WT) and Omicron B.1.1.529 and BA.2 variants using 72 longitudinal samples from 6 randomly selected individuals. All samples tested showed no neutralization activities (ID_50_ = 15) against all 3 strains following the first dose of CoronaVac vaccine (Figure 2A). Neutralization activities were markedly improved following the second dose of vaccination as most of the individuals (5/6) showed significantly increased ID_50_, ranging from 35 to 128, against ancestral SARS-CoV-2 (Figure 2B). Nearly all individuals showed poor plasma neutralizations against Omicron B.1.1.529 and BA.2 variants following the second vaccine dose (Figure 2B). Although half of the individuals (3/6) maintained their plasma neutralizing activities against ancestral (WT) SARS-CoV-2 strain, the overall ID_50_ waned sharply 6 months after the second dose of CoronaVac vaccine (ID_50_ ranging 15–38) (Figure 2C). Interestingly, after a third boost of CoronaVac vaccine, both the efficacy and breadth of plasma neutralization were markedly improved (Figure 2D). Importantly, 4 out of 6 individuals developed an adequate level of cross-neutralizing activities against Omicron sublineages B.1.1.529 and BA.2 (Figure 2D), which was not seen in individuals prior to receiving their third dose of CoronaVac vaccine. In line with the evidence from mRNA vaccine–induced nAb protection, a third booster of CoronaVac vaccine was necessary to markedly increase the efficacy and breadth of the protective nAb response (16).

**Figure 3:**
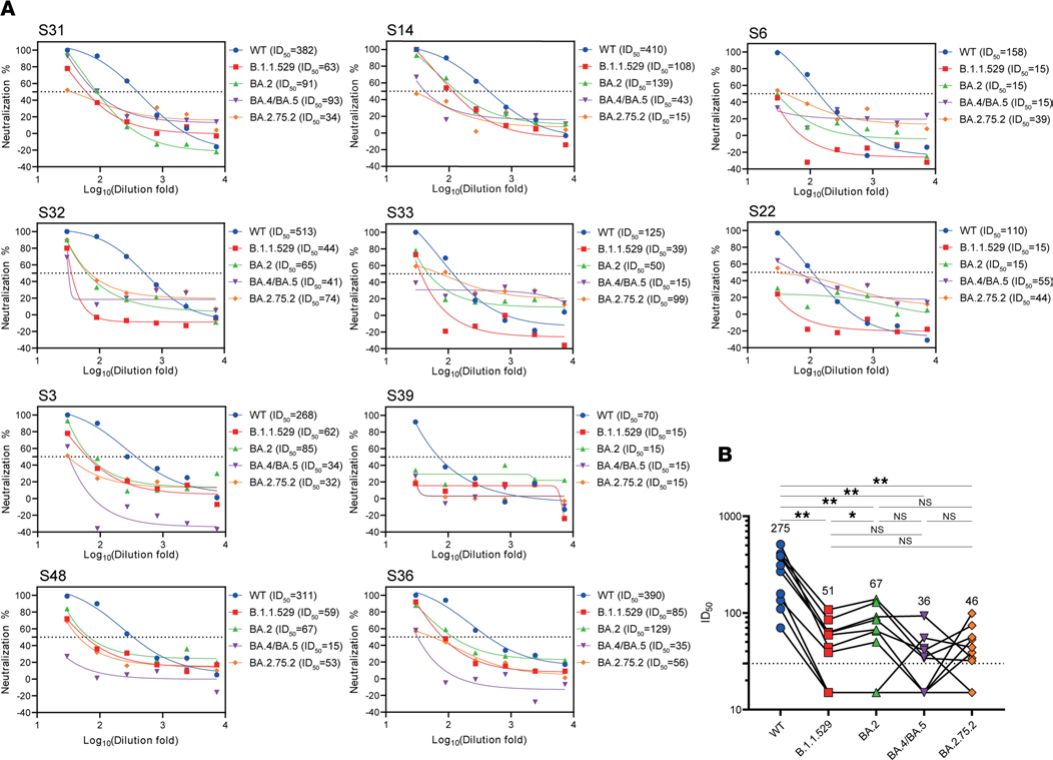
Protection of neutralizing antibody against SARS-CoV-2 variants after the third dose of CoronaVac. (**A**) Neutralizing activities against SARS-CoV-2 WT, B.1.1.529, BA.2, BA.4/BA.5, and BA.2.75.2 variants of plasma samples from 10 randomly selected individuals who received the third dose of CoronaVac vaccination. The horizontal dashed lines indicate 50% of the pseudovirus neutralization (ID_50_). Patient numbers and ID_50_ of different variants are shown at the top of each graph. (**B**) nAb titers (indicated as ID_50_) against SARS-CoV-2 WT, B.1.1.529, BA.2, BA.4/BA.5, and BA.2.75.2 variants measured after the third vaccination. Statistical significance was determined using Wilcoxon’s matched pairs signed ranks test for comparison between SARS-CoV-2 variants. *P < 0.05; **P < 0.01.

Little is known about the neutralization potential of emerging Omicron subvariants, such as BA.4/BA.5 and BA.2.75.2, by inactivated vaccines. We addressed this urgent question by testing samples from 10 individuals who received the third vaccine booster, and 4 out of 10 donors showed broad neutralization against all 4 Omicron subvariants (Figure 3A). Six out of 10 donors exhibited robust nAb response against BA.4/BA.5 (Figure 3A). Interestingly, plasma from 8 out of 10 donors neutralized the BA.2.75.2 (Figure 3A). ID_50_ of third booster–elicited antibodies against BA.4/BA.5 and BA.2.75.2 was lower than that against B.1.1.529 and BA.2 (Figure 3B). However, such reduction was relatively minor compared with the ID_50_ against the ancestral SARS-CoV-2 (Figure 3B), which is consistent with the most recent reports (17, 18). BA.2.75.2 is suggested to be more immune evasive than BA.5 (18). Our results imply that the third booster of CoronaVac vaccine generates modest but relatively broad antibody protection against the Omicron subvariants currently circulating.

### Polyclonal circulating CD4^+^ T cell response.

**Figure 4:**
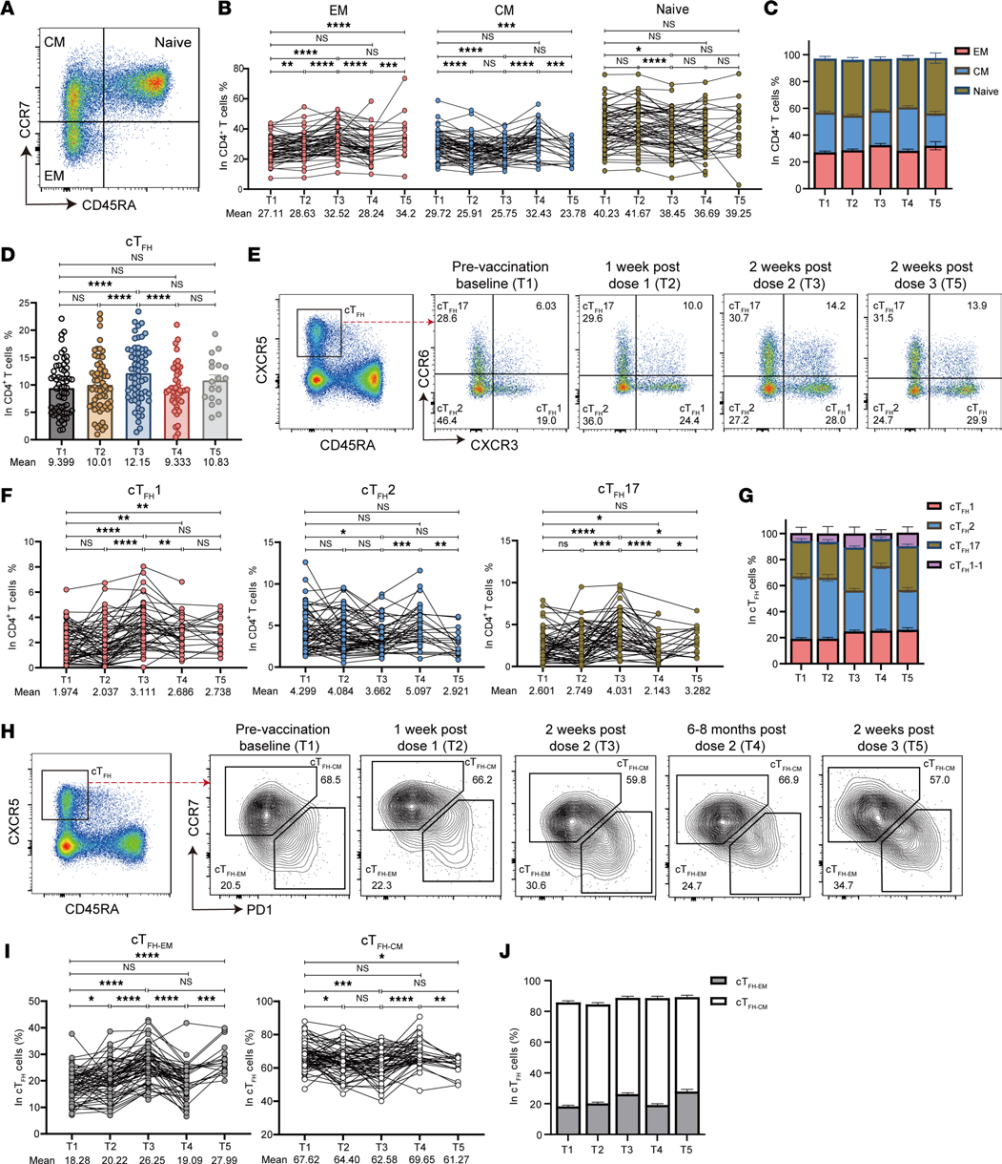
Characterization of polyclonal peripheral CD4^+^ T cells. PBMCs collected from vaccinated donors (*n* = 63) at 5 time points (T1–T5) were analyzed by 24-color flow cytometry. (**A**) Representative FACS plots of CD4^+^ T cell memory subsets and CD4^+^ naive T cells defined by CD45RA and CCR7. (**B**) Statistical analysis of the frequency of polyclonal effector memory (EM), central memory (CM), and naive CD4^+^ T cells at 5 time points. (**C**) Composition of polyclonal EM, CM, and naive CD4^+^ T cells from vaccinated individuals at 5 time points. Data are the same as in **B**. (**D**) Statistical analysis of the frequency of polyclonal cTfh cells at 5 time points. (**E**) Representative FACS diagrams of cTfh subsets grouped by CCR6 and CXCR3 at T1, T2, T3, and T5. (**F**) Longitudinal frequencies of polyclonal cTfh1, cTfh2, and cTfh17 cells measured by flow cytometry at 5 time points. (**G**) The proportion of cTfh1, cTfh2, cTfh17, and cTfh1–17 cells in polyclonal cTfh cells at 5 time points. Data are the same as in **F**. (**H**) Representative FACS plots of cTfh-EM and cTfh-CM cell subsets gated by PD-1 and CCR7 in cTfh cells at 5 time points. (**I**) Statistical analysis showing the differences of the frequencies of CCR7^hi^PD-1^–^ cTfh-CM and CCR7^lo^PD-1^+^ cTfh-EM cells at 5 time points. (**J**) The proportion of cTfh-EM and cTfh-CM cells in polyclonal cTfh cells at 5 time points. Data are the same as in **I**. Each dot represents an individual. Bars represent the mean values with SEM. Statistics were calculated using Wilcoxon’s matched pairs signed ranks test for comparison between time points (**B**, **D**, **F**, and **I**). *P < 0.05; **P < 0.01; ***P < 0.001; ****P < 0.0001.

Next, we set out to evaluate polyclonal peripheral CD4^+^ T cells in our longitudinal cohorts and determine how CD4^+^ T cell subsets might change by 3 doses of CoronaVac vaccine. Multicolor flow cytometry was used to measure the frequency of different CD4^+^ T cell subsets. The specific gating strategies based on the combination of signature surface molecules for lymphocytes were shown in Supplemental Figure 2A. Polyclonal memory and naive CD4^+^ T cells were identified based on CD45RA and CCR7 (Figure 4A) (19). Compared with the cells before vaccination (T1), we observed a marked increase of effector memory (EM) CD4^+^ T cells shortly after each dose of vaccination (T2, T3, T5), accompanied by decreased frequencies of central memory (CM) CD4^+^ T cells (Figure 4, B and C). Naive CD4^+^ T cell frequency was rather stable over our longitudinal follow-up (Figure 4, B and C). When evaluating the functional CD4^+^ T cell subsets, we found a relatively minor but significant increase of T helper 1 (Th1) and decrease of Th2 cells after the second dose (T3) compared with baseline (T1) yet negligible changes in Th17 cells (Supplemental Figure 2B). Tfh cells provide help to B cells to promote germinal center (GC) selection of memory and plasma B cells in health and disease (20–22). After the first dose of CoronaVac, we observed comparable circulating Tfh (cTfh) cells in vaccinees, while this frequency of polyclonal cTfh cells was significantly increased by a second dose of vaccine (Figure 4D). Polyclonal cTfh cell level was lowered over the following 6–8 months and maintained at a similar level upon a third boost of CoronaVac vaccine (Figure 4D). Interestingly, when measuring cTfh cell subsets featured by the differential expression of CXCR3 and CCR6 (7), we observed a marked change in the composition of these subsets following administration of a second and third dose of the vaccine (Figure 4E). With that being observed, the proportion of CXCR3-expressing cTfh1 cells were particularly increased by a second dose and third dose of vaccine, while the proportion of cTfh2 cells were largely reduced among cTfh cells (Figure 4F). Moreover, cTfh17 cells shared similar trends with those found in cTfh1 cells (Figure 4F). We also evaluated the total frequencies of each cTfh subset, and consistently, we observed increased cTfh1 cells by the second and third vaccination compared with baseline yet comparable levels or subtle differences with regard to the cTfh2 and cTfh17 cells (Figure 4G).

Next, we examined the effector and memory cTfh cell response following each vaccine dose, by looking into the surface expression of CCR7 and programmed cell death 1 (PD-1) on cTfh cells (7, 23). The frequency of CCR7^lo^PD-1^hi^ EM cTfh (cTfh-EM) cells was significantly increased by the second and third dose of vaccine (Figure 4, H and I). The frequency of cTfh-EM cells dropped markedly 6–8 months after the second dose of vaccination (Figure 4, I and J). Correspondingly, the frequency of CCR7^hi^PD-1^lo^ CM cTfh (cTfh-CM) cells increased significantly over the course of 6–8 months after the second vaccination (Figure 4, I and J). The kinetics and the rapid alterations of effector and memory cTfh cells following a vaccine administration further support cTfh cells as the key biomarker when evaluating the effectiveness and longevity of a vaccine response.

### Tfh1 cells represent the effector Tfh cells that effectively respond to the vaccination.

**Figure 5:**
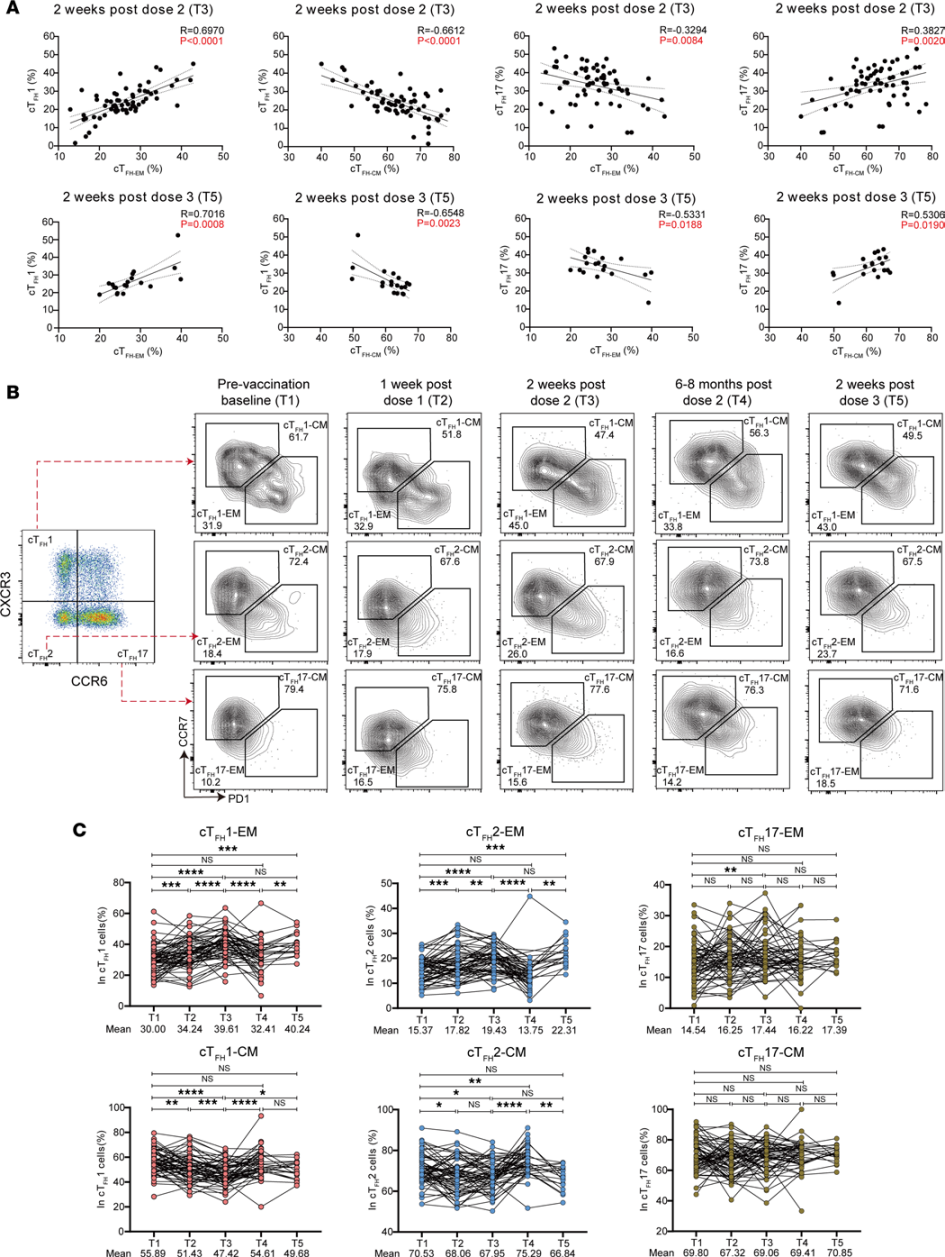
Characterization of effector and memory cTfh cells following vaccination. (**A**) Correlation analysis between cTfh1 and cTfh-EM/cTfh-CM cell frequencies; and between cTfh17 and cTfh-EM/cTfh-CM cell frequencies at 2 weeks after dose 2 (T3) and 2 weeks after dose 3 (T5). (**B**) FACS plots showing the representative cTfh1-EM, cTfh1-CM, cTfh2-EM, cTfh2-CM, cTfh17-EM, and cTfh17-CM cells gating from cTfh1, cTfh2, and cTfh17, cells by CCR7 and PD-1 at 5 time points. (**C**) Frequencies of cTfh1-EM, cTfh1-CM, cTfh2-EM, cTfh2-CM, cTfh17-EM, and cTfh17-CM cells within cTfh1, cTfh2, and cTfh17 cells at 5 time points. Each dot represents an individual. The 2-tailed Pearson’s correlation test was used (**A**). *P* and *R* values were indicated (**A**). Statistics were calculated using Wilcoxon’s matched pairs signed ranks test for comparison between time points (**C**). *P < 0.05; **P < 0.01; ***P < 0.001; ****P < 0.0001 (**C**).

Both cTfh1 and cTfh17 cells have been shown to correlate with antibody responses induced by SARS-CoV-2 infection or vaccination (24). However, how these Tfh cell subsets evolve and persist remains largely unknown. Human Th17 cells phenotypically resemble memory T cells in autoimmunity and antitumor response and show higher capacity for proliferative self-renewal and plasticity to interconvert into other CD4^+^ T cell subsets (25). By contrast, Th1 cells are more terminally differentiated during viral infection (26). To understand whether cTfh1 cells and cTfh17 cells possess different effector and memory potential, we performed Pearson’s correlation coefficient analysis between subsets of cTfh cells and effector/memory cTfh cells in donors after their second vaccination and third vaccine booster (Figure 5A). Strikingly, we found strong positive correlations between cTfh1 cells and cTfh-EM cells after both vaccinations. Similar positive correlations were noticed between cTfh17 cells and cTfh-CM cells (Figure 5A). In contrast, cTfh1 cells showed significant negative association with cTfh-CM cells, with the same trend between cTfh17 cells and cTfh-EM cells. To further validate the dominance of cTfh-EM cells in cTfh1 cells and of cTfh-CM cells in cTfh17 cells, we evaluated the frequency of EM and CM cells in each cTfh subset at 5 time points in 63 vaccinees (Figure 5B). We found that although more cTfh1 cells were CM cells before vaccination, markedly increased EM cells were observed in cTfh1 cells following the second and third vaccinations (from 30% to 39.61%, *P* < 0.0001; 30% to 40.24%, *P* = 0.0004) (Figure 5C). The proportion of EM cells sharply declined 6–8 months after the second vaccination (39.61% to 32.41%, *P* < 0.0001), suggesting a short-lived phenotype of cTfh1-EM cells. Nevertheless, a third booster of vaccine rapidly reinvigorated the frequency of cTfh1-EM cells to 40.24%. In contrast, over 70% of cTfh17 cells were CM cells, with only 10%–20% cTfh17 cells being EM cells over the course of 3 vaccinations (Figure 5C). Notably, the proportion of CM cells in cTfh17 cells remained intact over the course of 3 vaccine administrations, suggesting a relatively long-lived phenotype of cTfh17 cells (Figure 5C). Taken together, our data suggest that Tfh1 cells likely constitute the majority of effector Tfh cells that effectively respond to sequential vaccination, but they are short-lived. Conversely, Tfh17 cells may represent the most long-lived memory Tfh cells induced by the vaccine.

### Spike-specific CD4^+^ T cell response.

**Figure 6:**
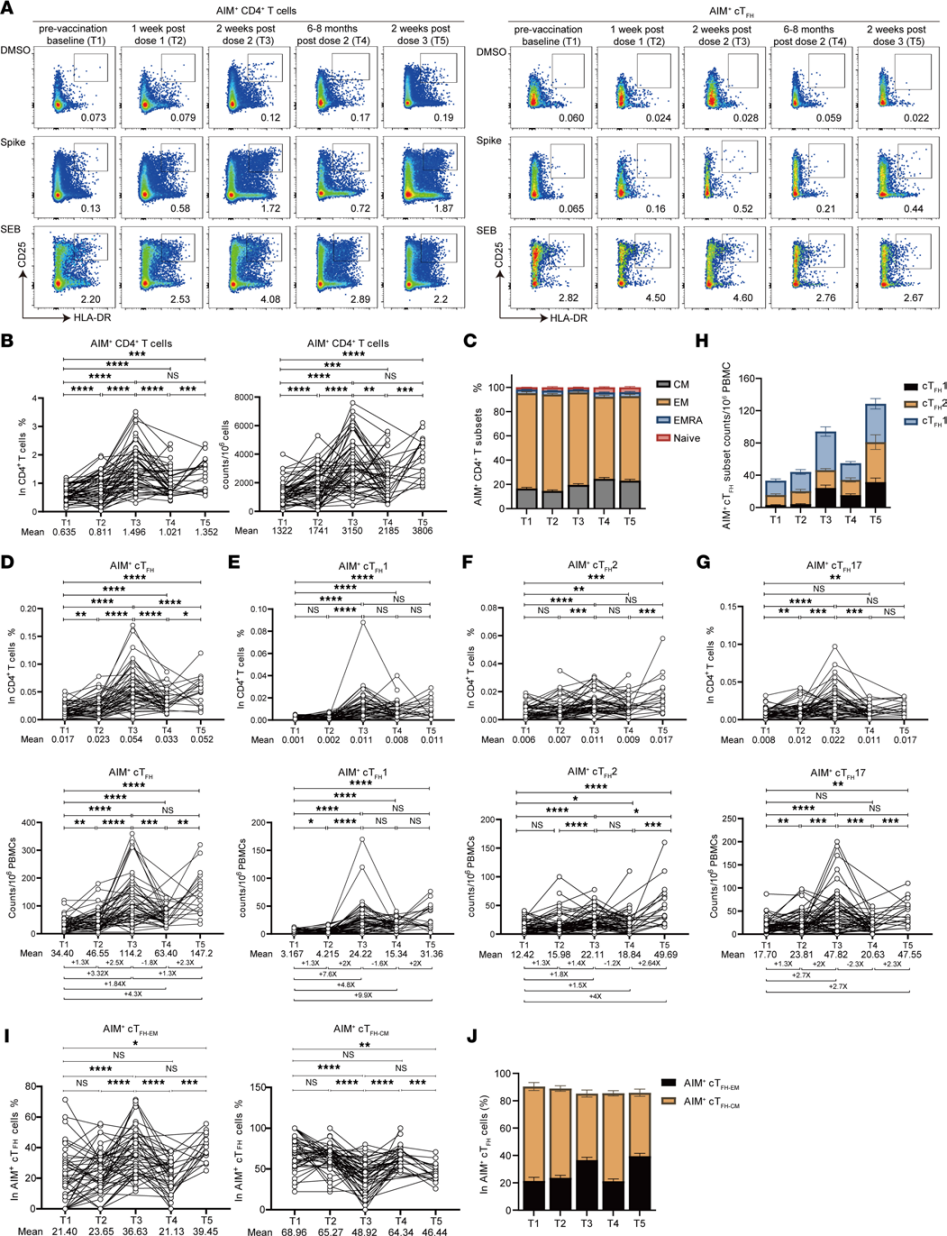
CoronaVac-induced spike-specific memory Tfh cells. PBMCs collected from vaccinated donors (*n* = 63) at 5 different time points (T1–T5) were ex vivo–stimulated with SARS-CoV-2 spike protein (S1+S2, 2 μg/mL, SinoBiological) in 5% CO_2_ at 37°C for 24 hours. SEB (1 μg/mL, Toxin Technology) was used as positive control. (**A**) Representative FACS plots of AIM^+^CD4^+^ T (HLA-DR^+^CD25^+^) cells and AIM^+^ cTfh (CXCR5^+^HLA-DR^+^CD25^+^) cells at 5 time points. The frequencies of AIM^+^CD4^+^ T cells (**B**), AIM^+^ cTfh cells (**D**), AIM^+^ cTfh1 cells (**E**), AIM^+^ cTfh2 cells (**F**), and AIM^+^ Tfh17 cells (**G**) were shown by the percentage in total CD4^+^ T cells and cell numbers in 10^6^ PBMCs. (**C**) The frequencies of AIM^+^ EM, CM, T cells that reexpress the naive cell marker CD45RA (TEMRA), and naive CD4^+^ T cells in AIM^+^CD4^+^ T cells at 5 time points. (**H**) The cell numbers of AIM^+^ cTfh1, cTfh2, and cTfh17 cells at 5 time points. Data are the same as in **E**–**G**. (**I**) Statistical analysis showing the alteration of the frequencies of AIM^+^ cTfh-EM and AIM^+^ cTfh-CM cells at 5 time points. (**J**) The proportion of AIM^+^ cTfh-EM and AIM^+^ cTfh-CM cells in AIM^+^ cTfh cells at 5 time points. Data are the same as in **I**. Each dot represents an individual participant. Bars represent the mean values with SEM. Statistics were calculated using Wilcoxon’s matched pairs signed ranks test for comparison between time points (**B** and **D**–**G**). *P < 0.05; **P < 0.01; ***P < 0.001; ****P < 0.0001.

To determine whether CoronaVac inactivated vaccine can induce durable antigen-specific memory CD4^+^ T cell responses, we utilized activation-induced marker (AIM) assay and evaluated the SARS-CoV-2 spike-specific response. PBMCs were stimulated with SARS-CoV-2 spike peptides, containing a pool of both S1 and S2 peptides, or staphylococcal enterotoxin B (SEB) as positive control. AIM^+^CD4^+^ T cells were defined by dual expression of CD25 and HLA-DR (Figure 6A) (27–29). To determine spike-specific CD4^+^ T cell responses from each time point, we also treated PBMCs with DMSO to define the spike-positive population (Figure 6A). Full gating strategies are provided in Supplemental Figure 3. The frequency of AIM^+^CD4^+^ T cells increased slightly after the first dose of vaccine (T2), with around 2-fold further increase after the second dose of vaccine (T3), compared with that at baseline (T1) (Figure 6, A and B). These results indicate robust induction of SARS-CoV-2 spike-specific CD4^+^ T cell responses after vaccination. Moreover, 6–8 months after the second vaccine dose (T4), the frequency of AIM^+^CD4^+^ T cells was sharply deceased about 1.4-fold compared with that of 2 weeks after the second vaccination (T3). The AIM^+^CD4^+^ T cells were maintained at a detectable level and higher than that at baseline (Figure 6B). As expected, a third booster of CoronaVac reinvigorated the AIM^+^CD4^+^ T cells to a level comparable to that soon after the second vaccination (T3) (Figure 6B). The proportion of EM cells in the total spike-specific CD4^+^ T cells were maintained at a high level at T2, T3, T4, and T5, while the proportion of CM cells increased substantially at 6–8 months after the second dose (T4) (Figure 6C).

To further assess the functionality of CoronaVac-induced CD4^+^ T cell responses, we characterized the SARS-CoV-2 spike-specific circulating Tfh cells (CXCR5^+^HLA-DR^+^CD25^+^CD4^+^) (Figure 6A). Notably, although the virus-specific IgG and nAbs were low to nondetectable 1 week after the first vaccination (T2), we found slightly increased frequency of spike-specific cTfh cells relative to the baseline (*P* = 0.0043). Similar to the total spike-specific CD4^+^ T cells, the frequency and number of spike-specific cTfh cells were further increased after the second dose of vaccine but steeply decreased 6–8 months later (Figure 6D). The third dose of CoronaVac vaccination enhanced the spike-specific cTfh response to a similar magnitude shortly after a second vaccine booster (Figure 6D). Spike-specific cTfh cells were further examined to understand the memory potential and superiority of their functional subsets, which might support the humoral immune response differently. Interestingly, we found that the magnitude of spike-specific cTfh1 response was relatively constant after boost by a dose of CoronaVac vaccine, with no significant decrease or increase of cTfh1 cells over 6–8 months, and by a third booster (Figure 6, E and H). Interestingly, the highest spike-specific cTfh2 cell numbers were found after a third boost of vaccine (Figure 6, F and H), which we did not observe in polyclonal cTfh cells (Figure 4F). Spike-specific cTfh17 cells, however, shared a similar kinetics to polyclonal cTfh17 cells, where the second dose of vaccine significantly mounted spike-specific cTfh17 cell response, which declined over 6–8 months but was reinvigorated by a third vaccine booster (Figure 6, G and H). Of note, when evaluating the memory potential of spike-specific cTfh cells by CCR7 and PD-1, we found significantly increased frequency of spike-specific CCR7^lo^PD-1^hi^ cTfh-EM cells by the second dose (Figure 6I). Similar to the polyclonal Tfh cell response shown in Figure 4I, the frequency of spike-specific cTfh-EM cells waned 6–8 months after the second dose of vaccination but was reinvigorated by the third dose of vaccine booster (Figure 6I). Correspondingly, spike-specific cTfh-CM cells were markedly reduced by the second or the third vaccination (Figure 6, I and J). Together, these data suggest that the second dose and third dose of CoronaVac vaccine induced robust spike-specific CD4^+^ T cell and cTfh cell responses, with different functional subsets sharing distinctive patterns.

**Figure 7:**
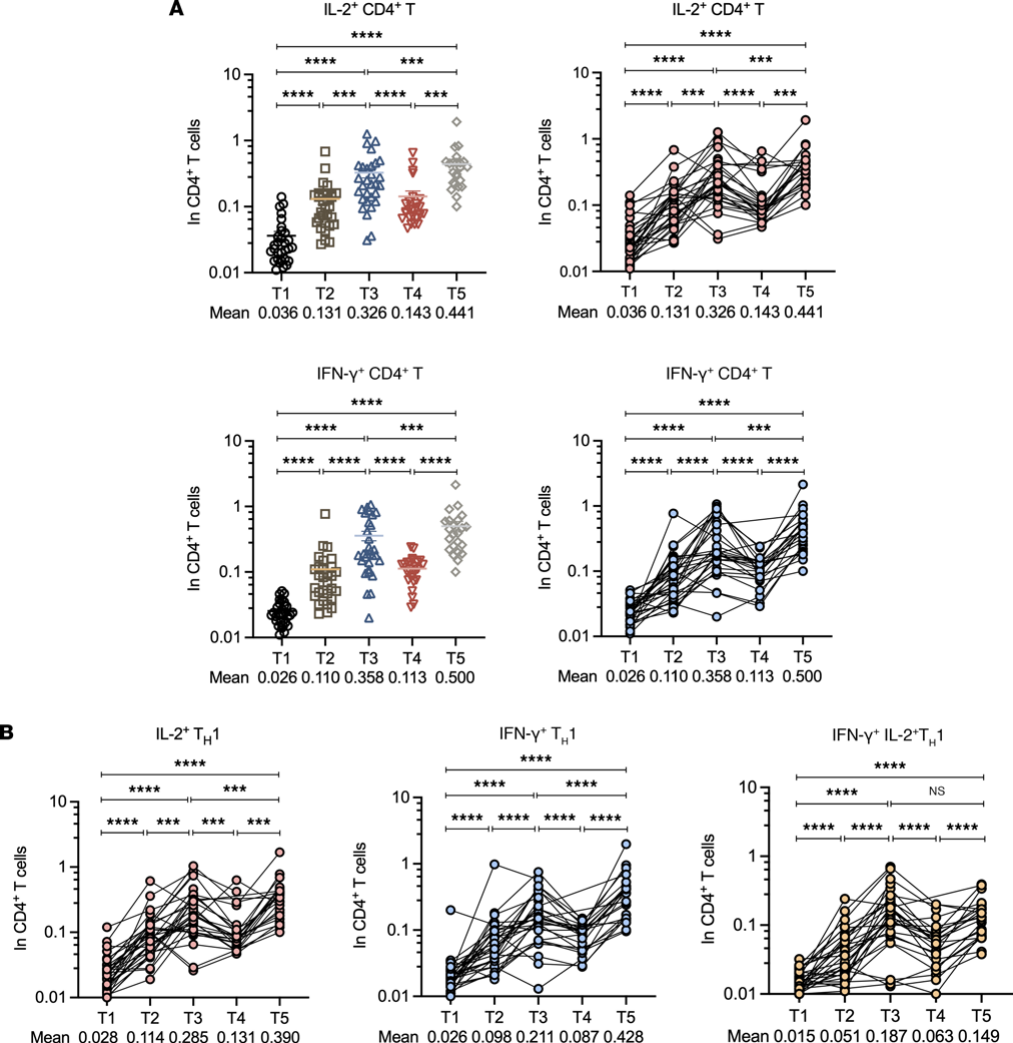
Cytokine-producing spike-specific CD4^+^ T cell responses. PBMCs collected from vaccinated donors at 5 different time points (T1–T3, *n* = 30; T4, *n* = 25; T5, *n* = 23) were ex vivo–stimulated with SARS-CoV-2 spike protein (S1+S2, 2 μg/mL, SinoBiological) in 5% CO_2_ at 37°C for 24 hours. (**A**) Frequencies of IL-2^+^ (top) and IFN-γ^+^ (bottom) spike-specific CD4^+^ T cells detected after SARS-CoV-2 peptide stimulation at different time points. (**B**) Frequencies of IL-2^+^ (left) and IFN-γ^+^ (middle), and IL-2^+^ + IFN-γ^+^ (right) spike-specific Th1 cells observed following SARS-CoV-2 peptide stimulation at different time points. Each dot represents an individual. Statistics were calculated using Wilcoxon’s matched pairs signed ranks test for comparison between time points (**A** and **B**). ***P < 0.001; ****P < 0.0001.

In line with the spike-specific CD4^+^ T cell response, markedly increased spike-specific IL-2– and IFN-γ–producing CD4^+^ T cells were noticed 7 days after CoronaVac vaccination (Supplemental Figure 4A and Figure 7A). The second and third CoronaVac booster further increased the frequencies of IL-2^+^CD4^+^ and IFN-γ^+^CD4^+^ T cells relative to baseline and the first immunization (Supplemental Figure 4, B and C, and Figure 7A). We further assessed the cytokine production from spike-specific Th1 cells by gating on the CXCR3^+^CD4^+^ T cells and found similar trends of IL-2 and IFN-γ production promoted by multiple CoronaVac vaccinations (Figure 7B). These data, together, indicate that the second and third homologous CoronaVac boosters elicit functional spike-specific CD4^+^ T cells necessary for regulating antiviral responses.

### Correlations between CD4^+^ T cells and antibody responses following CoronaVac vaccination.

To determine the relationship between CD4^+^ T cells and the production of SARS-CoV-2 antibodies after CoronaVac vaccination, we first analyzed the polyclonal circulating CD4^+^ T subsets and SARS-CoV-2 antibodies after the second and third vaccine doses (T3 and T5). The correlation matrix analysis using nonparametric Spearman’s rank test revealed no statistical significance on the correlation between polyclonal cTfh cells and antibody titers after the second dose (T3). Other CD4^+^ T cell subsets also showed negligible associations (Supplemental Figure 5A, left). By contrast, polyclonal cTfh cells were positively correlated with both SARS-CoV-2 spike-specific IgG (*R* = 0.4876, *P* = 0.0401) and IgM (*R* = 0.5145, *P* = 0.0289) after the third dose of CoronaVac vaccine (T5) (Supplemental Figure 5A, right, and Supplemental Figure 5B). Total cTfh cells and the level of SARS-CoV-2 nAb showed borderline correlations (*R* = 0.3765, *P* = 0.0618) (Supplemental Figure 5B). Interestingly, we found that both polyclonal cTfh1 (*R* = 0.4947, *P* = 0.0369) (Supplemental Figure 5C) and cTfh17 (*R* = 0.5679, *P* = 0.0140) (Supplemental Figure 5E) cells were positively correlated with virus-specific IgM after the third dose (T5), while there was no correlation for cTfh2 cells (Supplemental Figure 5D).

**Figure 8:**
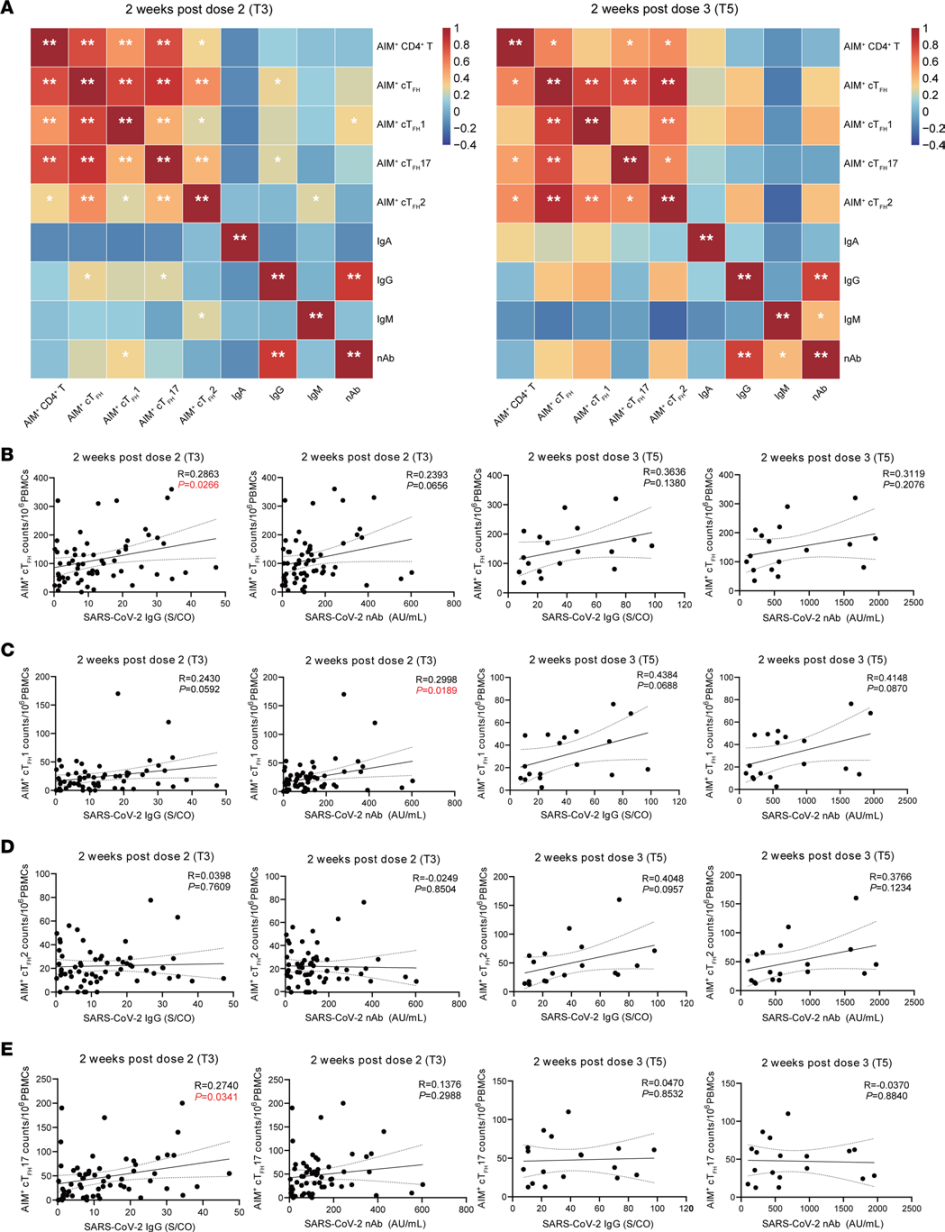
Correlations between CD4^+^ T cells and antibody responses following CoronaVac vaccination. (A) The correlation matrix analysis shows the correlation between AIM^+^CD4^+^ T cell subsets and SARS-CoV-2 antibodies after the second (T3) and third (T5) dose. Red shows positive correlation; blue represents negative correlation. The color intensity shows the proportion to the correlation coefficients. Correlation analysis between AIM^+^ cTfh (B), AIM^+^ cTfh1 (C), AIM^+^ cTfh2 (D), and AIM^+^ cTfh17 (E) cell numbers and SARS-CoV-2–specific IgG and nAb titers at T3 and T5. Each dot represents an individual. The 2-tailed, nonparametric Pearson’s and Spearman’s rank correlation tests were used, and results corrected after both analyses are shown (A–E). *P < 0.05; **P < 0.01 (A). *P* and *R* values were indicated (B–E).

Next, we evaluated the relationship between spike-specific AIM^+^CD4^+^ T cell subsets and SARS-CoV-2 antibody titers after the second dose (T3) and third dose of vaccines (T5) (Figure 8A). After multiple corrections with both Spearman’s rank correlation coefficient test and Pearson’s correlation coefficient test, we found no correlation between AIM^+^CD4^+^ T cell numbers and virus-specific antibody titers at T3 and T5 (Figure 8A). Interestingly, there was a positive correlation between spike-specific cTfh numbers and SARS-CoV-2 IgG (*R* = 0.2863, *P* = 0.0266) and nAb (*R* = 0.2393, *P* = 0.0656) titers after the second dose (T3) (Figure 8B). Similar trends were also found after the third dose (T5), but the results were not statistically different (Figure 8B). In addition, we observed that spike-specific CXCR3^+^ cTfh1 cell numbers positively associated with spike-specific IgG (*P* = 0.0592) and nAb titers (*P* = 0.0189) at T3 (Figure 8C). The trends still held at T5, although they were not statistically significant (Figure 8C). By contrast, there were no correlations between the number of spike-specific cTfh2 cells and IgG and nAb levels after the second (T3) and third (T5) dose (Figure 8D). It is worth mentioning that AIM^+^ cTfh17 cell numbers were positively correlated with SARS-CoV-2 IgG (*R* = 0.2740, *P* = 0.0341) after the second dose (T3) but not after the third dose (Figure 8E). Together, our results suggest that the second and third doses of CoronaVac vaccine induce spike-specific cTfh cells closely associated with serum antibody response and are capable of supporting humoral immune response.

## Discussion

More than 2 billion doses of CoronaVac have been administered in more than 40 countries (30). Recent evidence has shown that a homologous third dose of CoronaVac is associated with further increased SARS-CoV-2–specific antibodies (2–4). Although nAb titers were found to be lower in vaccinees who received a third booster dose of CoronaVac compared with vaccinees who received 3 doses of mRNA vaccines (31), in general, the second and third doses of CoronaVac were effective in preventing COVID-19–related mortality (74.8% for those aged >65, 80.7% for those aged 50–64, 82.7% for those aged 18–51) and severe complications (58.9% for those aged >65, 67.1% for those aged 50–64, 77.8% for those aged 18–51) (32). Consistent reductions in risk are observed with a third booster dose of CoronaVac (31–34). However, it remains unclear how a third CoronaVac vaccine dose affects the magnitude and quality of immune responses, particularly against the highly divergent variant Omicron. Here, we longitudinally evaluated the CoronaVac vaccine–elicited antibody and CD4^+^ T cell responses for 300 days. This allowed us to fill the knowledge gap of whether the inactivated SARS-CoV-2 vaccine may induce persistent and high-quality humoral immune response against Omicron subvariants, which have been substantially addressed by mRNA vaccine platforms.

It has been shown that 2 doses of mRNA vaccine induce robust and durable antibody response lasting for 6~9 months (35, 36). Different from the mRNA vaccine, nAb titers elicited by the second CoronaVac vaccine waned rapidly from the peak levels. Most of the individuals (70/83) displayed no detectable nAb titers 6~8 months after the second dose. Nevertheless, a third dose of CoronaVac vaccine significantly reinvigorated nAb responses. In particular, a third vaccine dose substantially improved the neutralization activities against Omicron B.1.1.529 and BA.2 variants. Of note, we found that the levels of SARS-CoV-2 IgG and nAb were negatively correlated with the age of participants at 2 weeks after the third dose, indicating elderly people may poorly respond to the CoronaVac vaccine and may require a fourth booster.

In our longitudinal study of vaccinated individuals, we found robust SARS-CoV-2 spike-specific memory CD4^+^ T cell responses following a second and third dose of CoronaVac vaccine in most of the participants. Moreover, we found that CoronaVac vaccination markedly altered the frequencies of polyclonal peripheral CD4^+^ T cell subsets, including a marked increase in the frequency of Th1 cells and the changes among cTfh subsets. SARS-CoV-2 mRNA vaccines can induce robust antigen-specific Tfh responses in both peripheral blood and lymph nodes that are maintained for 6 months (8, 14, 15). Similarly, our study found that CoronaVac vaccine efficiently elicited spike-specific IFN-γ/IL-2–producing CD4^+^ T cells and cTfh cells necessary for the antiviral and antibody response (24, 37–39). The expanded spike-specific cTfh cells were biased toward the proinflammatory cTfh17 subsets after the second and third dose of CoronaVac, which was also found after SARS-CoV-2 infection (40, 41). Interestingly, we found that circulating Tfh1 cells were positively associated with effector CCR7^lo^PD-1^hi^ cTfh cells. These EM-like Tfh cells are known to indicate the Tfh cell activity in secondary lymphoid organs and effectively respond to the vaccination (23). We further found EM-like CCR7^lo^PD-1^hi^ proportion of Tfh1 cells were particularly sensitive to the antigen and were rapidly boosted following the second and third CoronaVac vaccination. In contrast, cTfh17 cells were highly enriched with CM-like CCR7^hi^PD-1^lo^ cTfh-CM cells. The frequency of cTfh17-CM cells remained stable over the course of the administration of 3 vaccinations. Similar to the bulk cTfh cells, the frequency of spike-specific cTfh-EM cells was markedly invigorated by the second and third dose of vaccine. These results further support the notion that cTfh-EM cells may serve as a reliable biomarker when evaluating the effectiveness of vaccine-induced humoral immune response. Targeting cTfh-EM cells may also improve the vaccine response, which is worth investigating in future studies.

The positive correlation between cTfh1 cells and SARS-CoV-2 IgM and IgG titers was reported in COVID-19 convalescent individuals (7, 42) and in other infections (43–45). This evidence, coupled with our data herein, inspired us to interrogate whether the cTfh cell subsets associated with spike-specific antibody response. Indeed, clear correlations existed between the polyclonal cTfh cells and SARS-CoV-2 IgG and IgM antibody titers induced by a third dose of CoronaVac vaccine. A strong positive correlation between polyclonal cTfh1 cells and IgM was also found. Interestingly, we also observed a positive correlation between the cell numbers of spike-specific cTfh cells, cTfh1 cells, and SARS-CoV-2 IgG and nAb titers 2 weeks after the second and third vaccination. In brief, our data demonstrated that CoronaVac-induced Tfh responses were highly related to high-affinity antibody responses.

It should be noted that a more striking relationship between the Tfh cell and humoral immune response was observed at T3 compared with T5. A possible explanation for this is the difference in sample size. We were able to collect more longitudinal samples at T3 than at T5, which may have resulted in a clearer statistical relationship at T3. Biologically speaking, at T3, vaccine recipients received a more classical prime-boost immunization where the GC response reaches its peak. In response to a foreign antigen, a robust Tfh cell–GC B cell coordination is formed in a relatively clean system. At T5, a more complex GC response was induced, where a mixed GC response with a few long-lasting GCs and new GCs could be present in the same vaccinee. The phenomenon of “original antigenic sin” (46) could also impact the recruitment of new B cell clones into the GC response after repeated exposure to the same antigen, and how this phenomenon affects the Tfh cell response is unknown. It is highly likely that memory Tfh cells would compete with the new Tfh clones in providing help to the GC B cells, which may contribute to a less clear relationship between bulk Tfh cells and humoral immune response as the “help-kinetics” from memory Tfh cells and newly activated Tfh cells may be different after a third exposure to the same T-dependent antigen.

Sequential COVID-19 vaccinations with diverse vaccine platforms can effectively induce robust adaptive immune responses that provide protection against severe complications caused by SARS-CoV-2 and its subvariants (24, 37, 47). Although the magnitudes of spike-specific and variant-specific antibody and T cell responses are mostly comparable between BNT162b2 and mRNA-1273, and higher than those induced by Ad26.COV2.S and NVX-CoV2373, direct comparison studies on comprehensive clinical and immunological parameters between inactivated COVID-19 vaccines such as CoronaVac and other vaccine platforms are limited (48–50). In general, inactivated vaccines like CoronaVac elicit relatively lower seropositivity and anti-spike receptor binding domain IgG antibody responses compared with mRNA vaccines like BNT162b2, and such antibody titers tend to wane faster than those induced by mRNA vaccines (51). Nevertheless, our study and others suggest that a third dose of CoronaVac can increase the overall and nAb titers (Figure 1), potentially narrowing the quantitative gap of antibody titers between CoronaVac and mRNA vaccines (52). Interestingly, in line with some recent studies (53, 54), our data suggested that sequential administrations of CoronaVac induced robust effector and antigen-specific T cell response, similar to that elicited by BNT162b2, mRNA-1273, Ad26.COV2.S, and protein-adjuvanted vaccines such as NVX-CoV2373 (48–50). Moreover, our data indicate that a second and third CoronaVac vaccination effectively boosted antigen-specific Tfh cells, the key Th cells regulating antibody maturation, similar to mRNA vaccines (8, 49). Notably, we further identified that a CXCR3-expressing subset of Tfh cells, Tfh1 cells, represented the Tfh-EM cells, while Tfh17 cells assembled the CM-like Tfh cells (Tfh-CM) in response to sequential vaccinations. Further studies are needed to investigate whether mRNA and other vaccine types elicit a similar Tfh cell response.

There are several limitations in this study. First, the number of individuals enrolled after the third dose of CoronaVac is relatively small. This is in part because some of the individuals were infected with other viruses, such as influenza virus or had received a vaccination for hepatitis B virus, and no longer eligible based on our recruitment requirements. Our study, nevertheless, revealed the dynamics of polyclonal and SARS-CoV-2 specific CD4^+^ T cell response following each dose of CoronaVac vaccine. Despite the adequate knowledge acquired on this matter from mRNA-vaccinated individuals, there is a big gap in our understanding of T cell response elicited by inactivated SARS-CoV-2 vaccine and how it may evolve longitudinally. Our study can provide necessary insights on this matter and guide the design of a novel vaccine regimen of inactivated vaccine against SARS-CoV-2 and viruses beyond it.

## Methods

### PBMC and plasma isolation.

Blood collection and processing were performed as previously described (24). Briefly, whole blood was collected in EDTA-2K tubes (BD Biosciences) and processed for PBMC and plasma isolation. EDTA-2K tubes were first centrifuged (450*g*, 5 minutes, 4°C), and the plasma was harvested for storage at –80°C until required. Samples were further diluted with PBS (1:1) and separated using the Ficoll-Hypaque (GE Healthcare Life Sciences, now Cytiva) density gradient (centrifugation 450*g*, 25 minutes, 20°C, without brake). PBMC layers were carefully collected and washed twice. After centrifugation, cells were resuspended in recovery media containing 10% DMSO (Gibco), supplemented with 10% heat-inactivated fetal bovine serum (FBS; Gibco). Aliquots of cells were quickly transferred to a freezing container (Corning) at –80°C overnight. Samples were stored in liquid nitrogen until further use.

### Immunophenotyping by flow cytometry.

Frozen aliquots of PBMCs were immediately thawed into prewarmed complete RPMI-1640 (Gibco) supplemented with 10% heat-inactivated FBS, carefully washed once, and resuspended in FACS buffer (PBS with 2% heat-inactivated FBS), with diluted 7-AAD (1:100 in the FACS buffer) added to exclude dead cells, followed by Fc receptor block (1:5 dilution, Miltenyi Biotec) to block nonspecific staining. Cells were then stained with a cocktail of monoclonal antibodies including CD45RA–Alexa Fluor 488 (1:100, HI100), CD3-AF532 (1:200, UCHT1), CD4–Brilliant Violet (BV) 750 (1:200, SK3), CD8a-BV570 (1:200, RPA-T8), TCRγδ-BV480 (1:100, B1), CD19–Super Bright 436 (1:100, HIB19), CXCR5-APC (1:50, J252D4), CD25-PE (1:50, clone M-A251), CCR7–PE-Cy7 (CD197) (1:50, clone 3D12), HLA-DR–APC/Fire 750 (1:100, clone L243), CD183 (CXCR3)-BV421 (1:100, clone G025H7), CD196 (CCR6)-BV605 (1:100, clone G034E3), CD279 (PD-1)–BV650 (1:50, clone EH12.2H7), and CD127–APC-R700 (1:100, clone HIL-7R-M21) (Supplemental Table 1). After incubation for 30 minutes at 4°C in the dark, cells were washed twice in FACS buffer and then resuspended in 200 μL FACS buffer. Cells were kept on ice until acquisition.

Detailed immune phenotyping of CD4^+^ T cells using 24-color flow cytometry was performed on Cytek Northern Lights with standardized configuration. Dead cells were routinely excluded from the analysis by staining with 7-AAD. For CD4^+^ T cells, EM (CD45RA^−^CCR7^−^), CM (CD45RA^−^CCR7^+^), and naive (CD45RA^+^CCR7^+^) cells can be defined. Within the CD25^+^ compartment, CD4^+^ T cells can be identified as Treg (CD25^+^CD127^lo^) and T follicular regulatory (Tfr) (CD25^+^CD127^lo^CXCR5^+^PD-1^+^) cells. Within the CD25^–^ compartment, CD4^+^ T cells can be divided into Tfh (CD45RA^−^CXCR5^+^), Th1 (CD45RA^−^CXCR5^−^CXCR3^+^CCR6^−^), Th2 (CD45RA^−^CXCR5^−^CXCR3^−^CCR6^−^), and Th17 (CD45RA^−^CXCR5^−^CXCR3^−^CCR6^+^) cells. Tfh cells can be further divided into Tfh1 (CXCR3^+^CCR6^−^), Tfh2 (CXCR3^−^CCR6^−^), and Tfh17 (CXCR3^−^CCR6^+^) cells. Data were analyzed with FlowJo software (Version 10).

### AIM T cell assay and intracellular staining assay.

Around 1 × 10^6^ cells per 200 μL were plated in 96-well, U-bottom plates with complete RPMI-1640 medium containing 5% heat-inactivated FBS. After resting overnight in the incubator at 37°C with 5% CO_2_, cells (1 × 10^6^) were stimulated with SARS-CoV-2 spike protein (S1+S2, 2 μg/mL, SinoBiological) in 5% CO_2_ at 37°C for 24 hours. Costimulatory anti-CD28 (1 μg/mL, BioLegend) and anti-CD49d (1 μg/mL, BioLegend) were added (Supplemental Table 1). SEB (1 μg/mL, Toxin Technology) was used as positive control, and an equimolar amount of DMSO was used as negative control. Antigen-specific CD4^+^ T (HLA-DR^+^CD25^+^) and antigen-specific Tfh (CXCR5^+^HLA-DR^+^CD25^+^) cells were defined by the AIM assay.

For intracellular staining assay, 1 × 10^6^ PBMCs were cultured in the presence of SARS-CoV-2 spike peptide pools (S1+S2, 2 μg/mL) for 24 hours at 37°C. Costimulatory anti-CD28 (1 μg/mL) and anti-CD49d (1 μg/mL) were added. In addition, SEB (1 μg/mL) was used as positive control, and an equimolar amount of DMSO was used as negative control. After 24 hours, Brefeldin A (1:1,000, eBioscience) was added to the culture for an additional 4 hours. After incubation, cells were washed and stained with Fixable Viability Dyes (eFluor 520, eBioscience) for 30 minutes at 4°C. Cells were then stained with a cocktail of monoclonal antibodies for cell surface staining with Fc block. After surface staining, cells were permeabilized and stained with intracellular antibodies against IFN-γ–PE-Cy7 (1:50, clone 4S.B3) and IL-2–APC-R700 (1:50, clone MQ1-17H12) for 30 minutes in the dark at room temperature. Cells were kept on ice until acquisition by Cytek Northern Lights flow cytometer.

### SARS-CoV-2 IgG/IgM/IgA antibody measurement.

The concentrations of plasma SARS-CoV-2 IgG (Autobio Diagnostics), IgM (Autobio Diagnostics), and IgA (Beijing Wantai Biological Pharmacy) were measured by chemiluminescent microparticle immunoassay kits, according to the manufacturer’s instructions. The assay is based upon the 2-step indirect method. Briefly, SARS-CoV-2 IgG/IgM/IgA present in the sample binds to the SARS-CoV-2 antigen–coated microparticles. Then, HRP-conjugated anti-human IgG/IgM/IgA followed by a chemiluminescent substrate was added into the reaction system, resulting in a chemiluminescent reaction. The resulting chemiluminescent reaction was measured as RLU, which was proportional to the amount of SARS-CoV-2 IgG/IgM/IgA in the samples. Results were evaluated by S/CO. Samples with S/CO values <1.00 are considered nonreactive (NR). Samples with S/CO values ≥1.00 are considered reactive (R).

### SARS-CoV-2 nAb measurement.

The concentrations of plasma SARS-CoV-2 nAb were tested by chemiluminescent microparticle immunoassay (Autobio Diagnostics), according to the manufacturer’s instructions. This assay is based upon the 1-step competitive method. The amount of SARS-CoV-2 nAb in the samples is measured from the RLU by means of the stored calibration data and determined automatically by the system software. Samples with values <30 AU/mL are NR; values ≥ 30 AU/mL are R.

### SARS-CoV-2 pseudovirus neutralization assay.

Pseudotyped HIV incorporated in different variants of SARS-CoV-2 spike proteins (Vazyme) were used to test the neutralizing activity of serum from vaccination recruits. The SARS-CoV-2 pseudoviruses bearing WT and B.1.1.529 and BA.2 spike proteins were provided by Vazyme. Serum samples were first heat-inactivated in a water bath for 30 minutes at 56°C and then serially diluted 3-fold with complete DMEM from 1:20 to 1:4,860 in 96-well, flat-bottom culture plates in a total volume of 150 μL. The cell control with only cells and the virus control (VC) with virus and cells were set up in each plate. The SARS-CoV-2 pseudotyped viruses were diluted to 2 × 10^4^ TCID_50_/mL in complete DMEM, and 50 μL diluted pseudotyped virus was added to each well and incubated for 1 hour at 37°C. The sample wells were finally diluted from 1:30 to 1:7,290. We adjusted the HEK293-ACE2 (Vazyme) cell concentration to 4 × 10^5^ cells/mL with complete DMEM, added 50 μL of cell suspension into all wells, and incubated for 48 hours at 37°C and 5% CO_2_. Finally, Bio-Lite Luciferase Assay System (Vazyme) was employed to measure the firefly luciferase activity, to obtain the nAb content of the sample. nAb titers were calculated as ID_50_ expressed as the dilution of serum that resulted in a 50% reduction of luciferase luminescence compared with a VC.

### Statistics.

Concentrations of SARS-CoV-2 anti-spike IgG, anti-spike IgM, anti-spike IgA, and nAb in vaccinated individuals between 5 time points were compared using Wilcoxon’s matched pairs signed ranks test. The nonparametric Mann-Whitney *U* test was used to compare the effects of different time intervals between the first and second dose on SARS-CoV-2 antibody levels. The frequencies of polyclonal peripheral CD4^+^ T and spike-specific CD4^+^ T cell subsets were calculated using Wilcoxon’s matched pairs signed ranks test for comparison between different time points. The 2-tailed, nonparametric Spearman’s rank correlation test and Pearson’s test were used to evaluate the correlations between CD4^+^ T cells and antibody responses following CoronaVac vaccination and the correlation between age and SARS-CoV-2 antibody titers. Statistical analysis was carried out using GraphPad Prism (V 9.2.0) software, and the correlation matrix mapping used R (V 3.6.3) software. *P* values are indicated with asterisks, and *P* < 0.05 was considered statistically significant.

### Study approval.

A total of 88 participants (health care workers) who received 2 or 3 doses of SARS-CoV-2 vaccination (CoronaVac) at Affiliated Hospital of Jiangnan University and The Fifth People’s Hospital of Wuxi were recruited in our study from February 2021, and the study was initially done before December 2021. The medical ethical committees of the Affiliated Hospital of Jiangnan University (LS2021004) and The Fifth People’s Hospital of Wuxi (2020-034-1) reviewed and approved the study. Participants included were healthy adults aged 18 to 70 years without evidence of preceding SARS-CoV-2 infection. All individuals were nonatopic and with no infectious diseases or autoimmune diseases. Blood samples were collected at the following time points: prevaccination baseline (T1), 1 week after the first dose (T2), 2 weeks after the second dose (T3), 6–8 months after the second dose (T4), as well as 2 weeks after the third dose (T5). Written informed consent was obtained from all participants before sample collection.

### Data availability.

Values for all data points found in graphs are in the Supporting Data Values file.

## Author contributions

PZ conceived and designed the study. CC, TJ, YD, and TZ performed the experiments. FG, YD, ML, and XL participated in scientific discussion and recruited the patients. CC, TJ, JJ, DS, and ZB analyzed the data. CC, TJ, and FG helped with the original illustrations and draft. PZ, CC, and TJ are co–first authors, and PZ, XL, and FG are co–senior authors; the order in which they are listed was determined by workload. PZ wrote and revised the manuscript and led the submission.

## Supplementary Material

Supplemental data

Supporting data values

## Figures and Tables

**Table 1:**
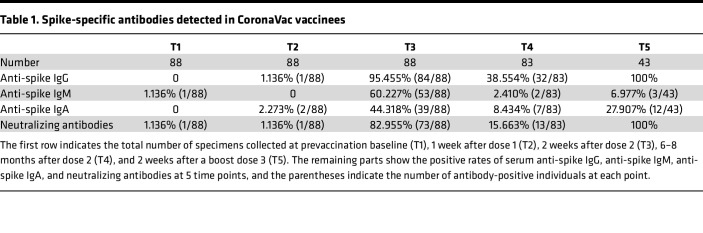
Spike-specific antibodies detected in CoronaVac vaccinees

## Version 1 06/29/2023

In-Press Preview

## Version 2 08/08/2023

Electronic publication
