# Supplementary material for: Longitudinal analysis of memory Tfh cells and antibody response following CoronaVac vaccination
Source: JCI Insight. 2023 Aug 8;8(15):e168437. doi: 10.1172/jci.insight.168437 (PMC10445683; doi:10.1172/jci.insight.168437)
Supplement: Supplemental data [file jciinsight-8-168437-s035.pdf]

Supplementary Figure

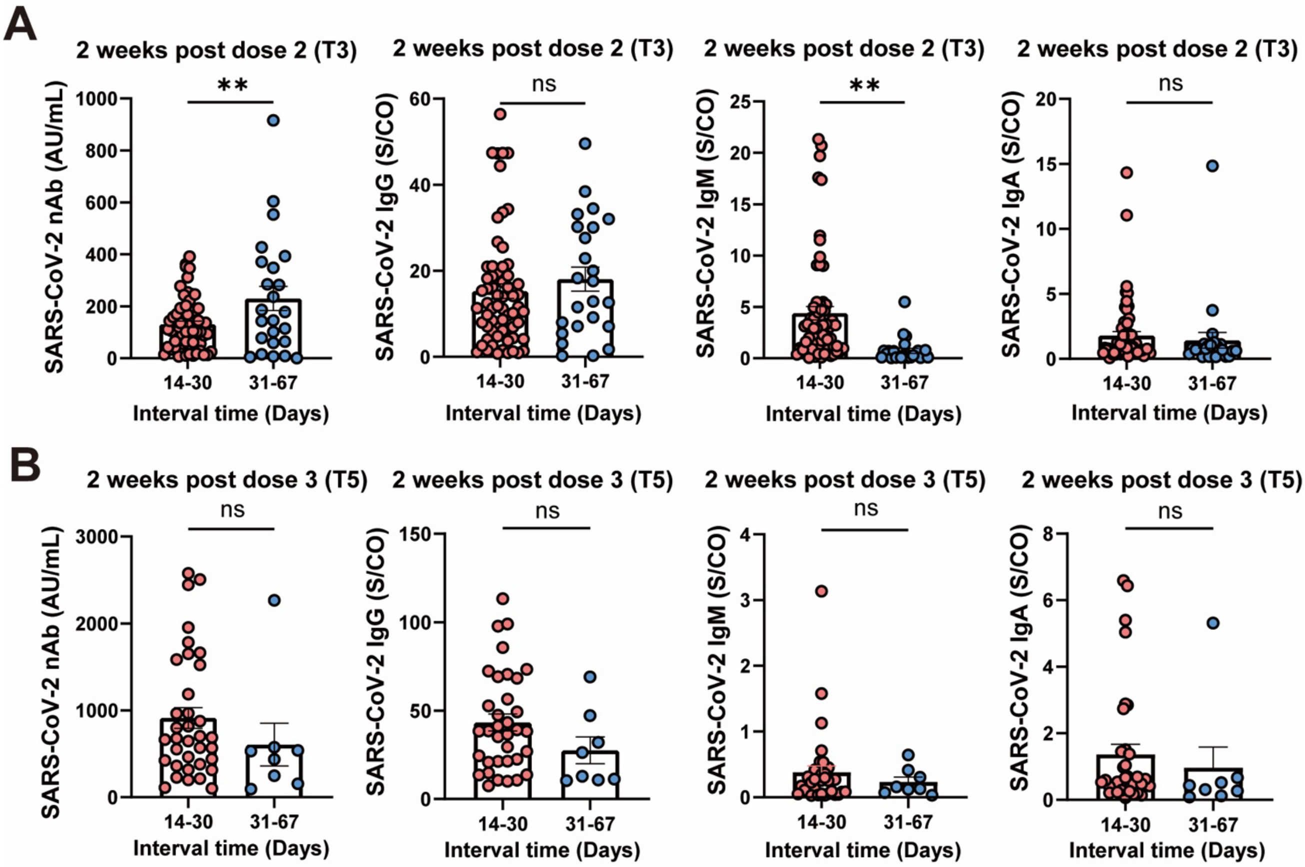

Supplementary figure S1: Vaccination interval and the levels of SARS-CoV-2-specific antibodies induced by CoronaVac vaccine

Effect of different time intervals between the primary and second dose on the levels of SARS-CoV-2 IgM, IgG, IgA and nAb at 2 weeks after the 2nd dose of vaccine (T3) (A) and 2 weeks after the 3rd dose of vaccine (T5) (B). Each dot represents an individual subject. Bars represent the mean values with SEM. Statistics were calculated using Mann-Whitney U test (A, B). *P < 0.05; **P < 0.01; ns, not significant.

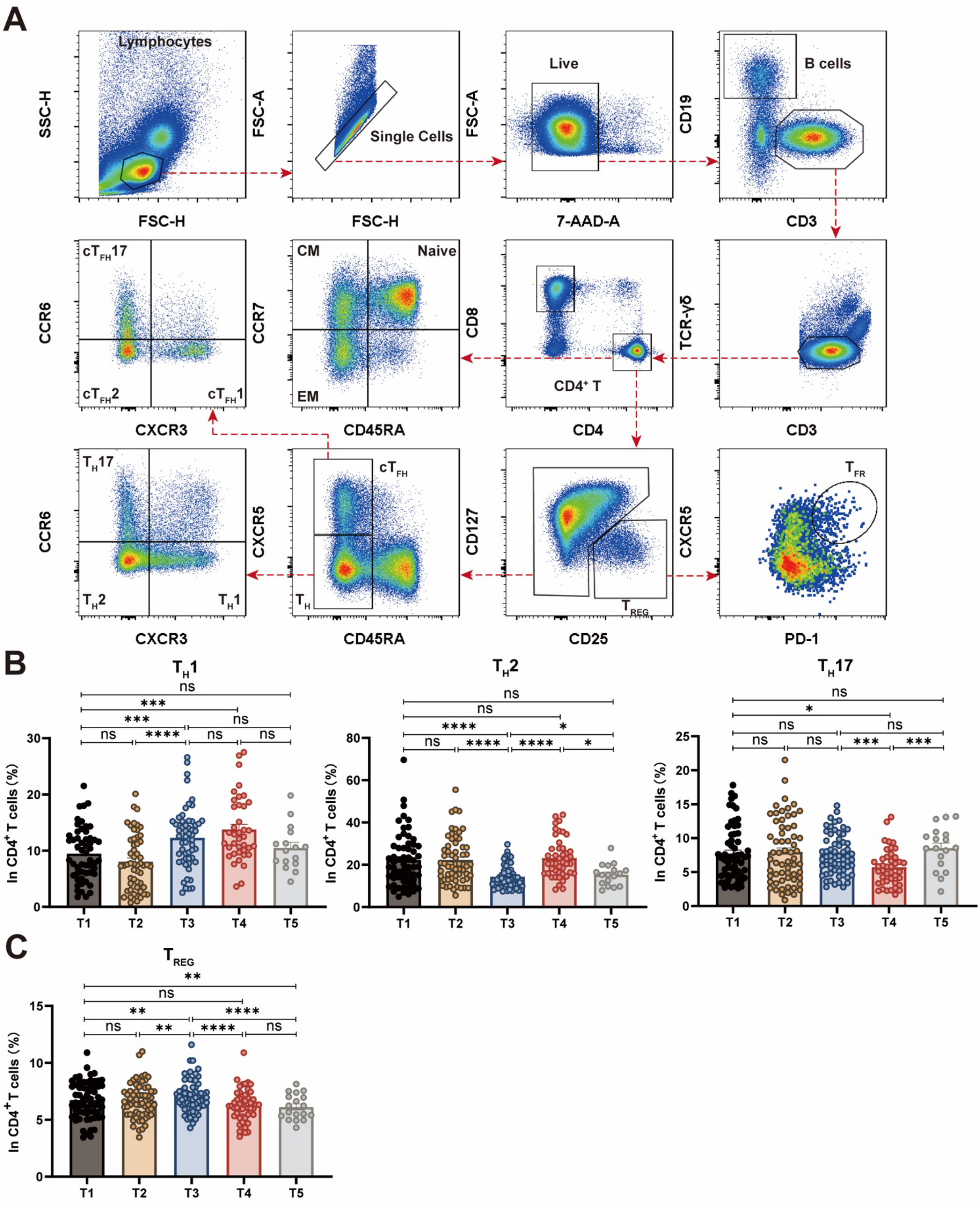

Supplementary figure S2: Polyclonal circulating CD4⁺ T cells.

(A) Representative plots illustrate the gating strategy of polyclonal CD4⁺ T cells. Lymphocytes were firstly gated, and doublets were excluded by FSC-H and FSC-A signals. Live cells were

identified by 7-AAD. $CD4^+$ T cells were gated on $CD3^+CD19^-TCR\gamma\delta^-$ cells. In particular, EM ($CD45RA^-CCR7^-$), CM ($CD45RA^-CCR7^+$) and naïve cells ($CD45RA^+CCR7^+$) are defined accordingly. T_{REG} ($CD25^+CD127^{low}$) and T_{FR} ($CD25^+CD127^{low}CXCR5^+PD\text{-}1^+$) cells are gated accordingly. CD25 negative cells can be divided into cT_{FH} ($CD45RA^-CXCR5^+$) cells and T_H ($CD45RA^-CXCR5^-$) cells. Based on the expression of CXCR3 and CCR6, cT_{FH} cells were further divided into $cT_{FH}1$ ($CXCR3^+CCR6^-$), $cT_{FH}2$ ($CXCR3^-CCR6^-$) and $cT_{FH}17$ ($CXCR3^-CCR6^+$) cells, while T_H cells were divided into T_H1 ($CXCR3^+CCR6^-$), T_H2 ($CXCR3^-CCR6^-$), and T_H17 ($CXCR3^-CCR6^+$) cells. Longitudinal dynamics of polyclonal T_H1, T_H2, T_H17 (B) and T_{REG} (C) cells during three doses of vaccines at five timepoints. Each dot represents an individual subject. Bars represent the mean values with SEM. Statistics were calculated using Wilcoxon matched-pairs signed rank for comparison between timepoints (B, C). *, $P < 0.05$; **, $P < 0.01$; ***, $P < 0.001$; ****, $P < 0.0001$; ns, not significant.

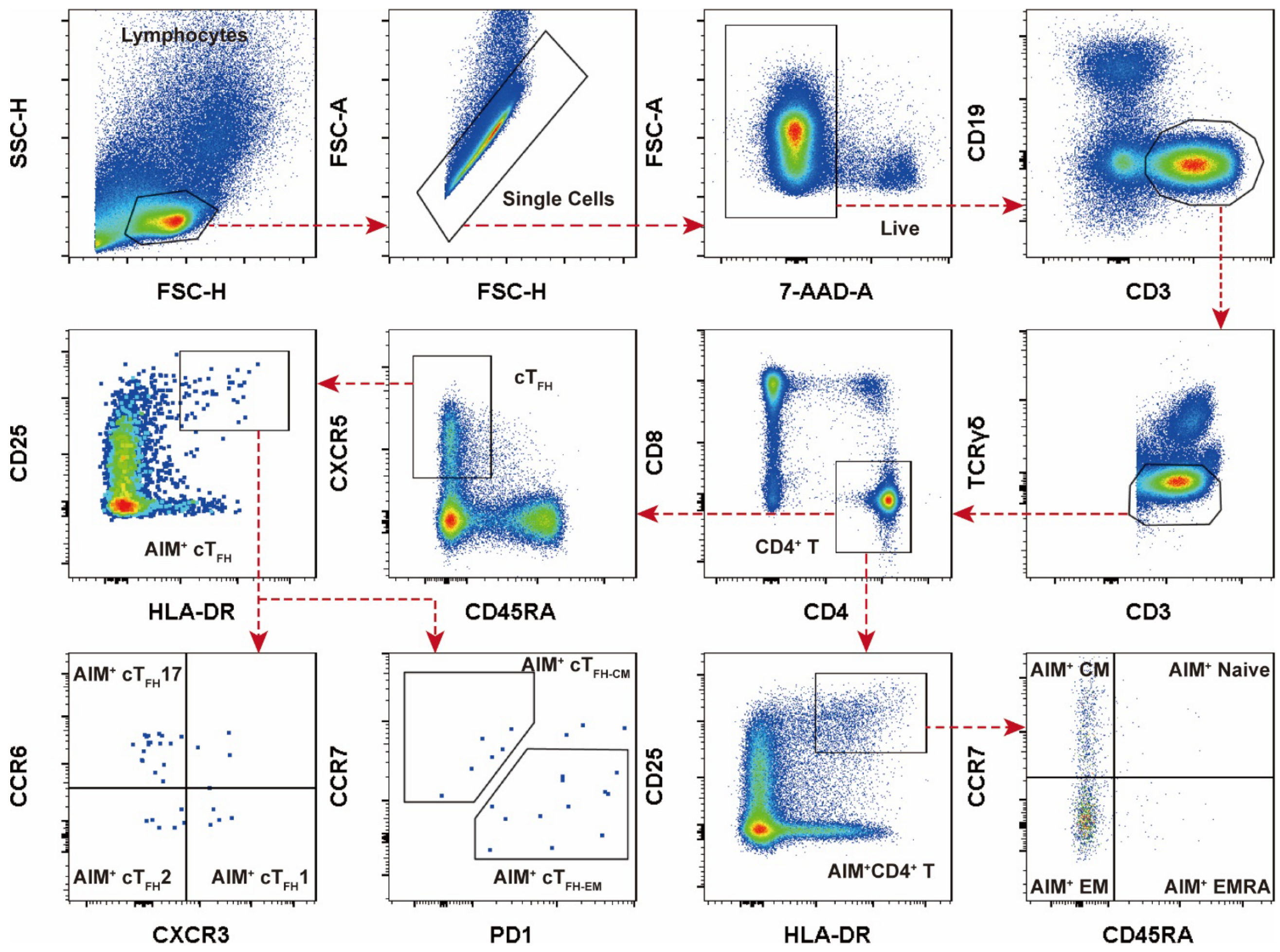

Supplementary figure S3: Gating-strategy for spike-specific CD4⁺ T and cT_{FH} cells.

PBMCs were stimulated with SARS-CoV-2 spike protein (S1+S2, 2 ug/mL, SinoBiological) for 24h. Representative plots illustrate the gating strategy. $CD4^+$ T cells were driven from $CD3^+CD19^-TCR\gamma\delta^-$ gating. Spike-specific $CD4^+$ T cells and spike-specific cT_{FH} cells were defined as $HLA\text{-}DR^+CD25^+CD4^+$ and $CXCR5^+$ $HLA\text{-}DR^+CD25^+CD4^+$, respectively. Spike-specific $CD4^+$ T cells were further divided into memory and naïve cells. Spike-specific cT_{FH} cells were further divided into $cT_{FH}1$, $cT_{FH}2$ and $cT_{FH}17$ cells according to the expression of surface molecules CXCR3 and CCR6, and divided into AIM^+ $cT_{FH\text{-}CM}$ and $cT_{FH\text{-}EM}$ cells by CCR7 and PD1.

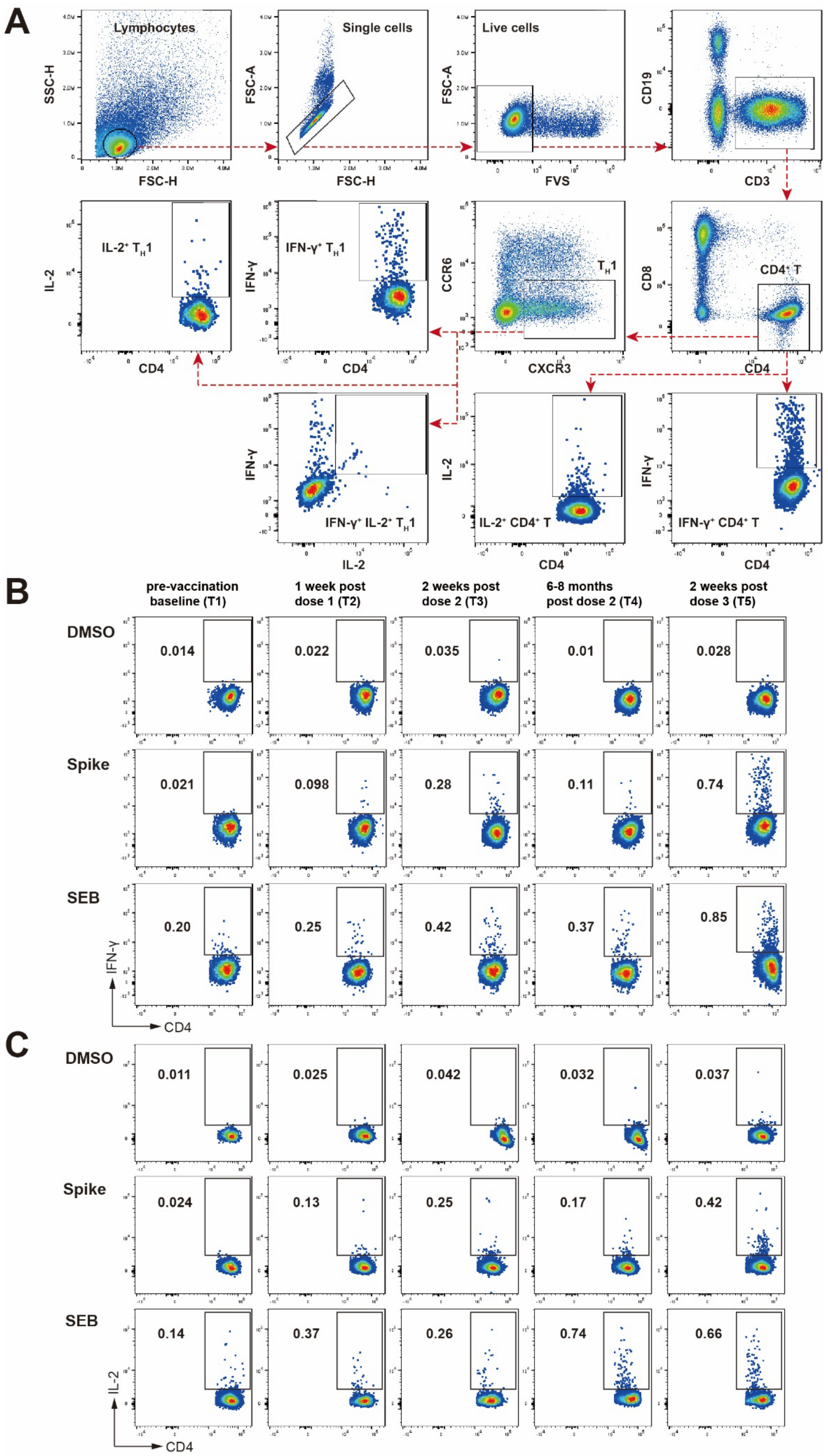
A
Lymphocytes
Single cells
Live cells
SSC-H
FSC-H
FSC-A
FVS
CD19
CD3
IL-2⁺ TH1
IFN-γ⁺ TH1
TH1
CD4⁺ T
IL-2
IFN-γ
CCR6
CXCR3
CD8
CD4
IFN-γ⁺ IL-2⁺ TH1
IL-2⁺ CD4⁺ T
IFN-γ⁺ CD4⁺ T
B
pre-vaccination baseline (T1)
1 week post dose 1 (T2)
2 weeks post dose 2 (T3)
6-8 months post dose 2 (T4)
2 weeks post dose 3 (T5)
DMSO
0.014
0.022
0.035
0.01
0.028
Spike
0.021
0.098
0.28
0.11
0.74
SEB
0.20
0.25
0.42
0.37
0.85
C
DMSO
0.011
0.025
0.042
0.032
0.037
Spike
0.024
0.13
0.25
0.17
0.42
SEB
0.14
0.37
0.26
0.74
0.66

Supplementary figure S4: Gating-strategy and representative plots for spike-specific $CD4^+$ T cells using ICS assay.

(A) Representative plots illustrate the gating strategy of spike-specific $CD4^+$ T cells. $CD4^+$ T cells were gating from live $CD3^+CD19^-$ lymphocytes. T_H1 cells were defined as $CXCR3^+$ $CCR6^-$ $CD4^+$ T cells. IFN-γ^+ $CD4^+$ T cells, IL-2^+ $CD4^+$ T cells, IFN-γ^+ T_H1, IL-2^+ T_H1 and IFN-γ^+ IL-2^+ T_H1 were further gated based on the cytokine production. Representative FACS plots of IFN-γ^+ $CD4^+$ T cells (B) and IL-2^+ $CD4^+$ T cells (C) were shown at five time points (T1-T5). DMSO and SEB were used as negative and positive controls, respectively.

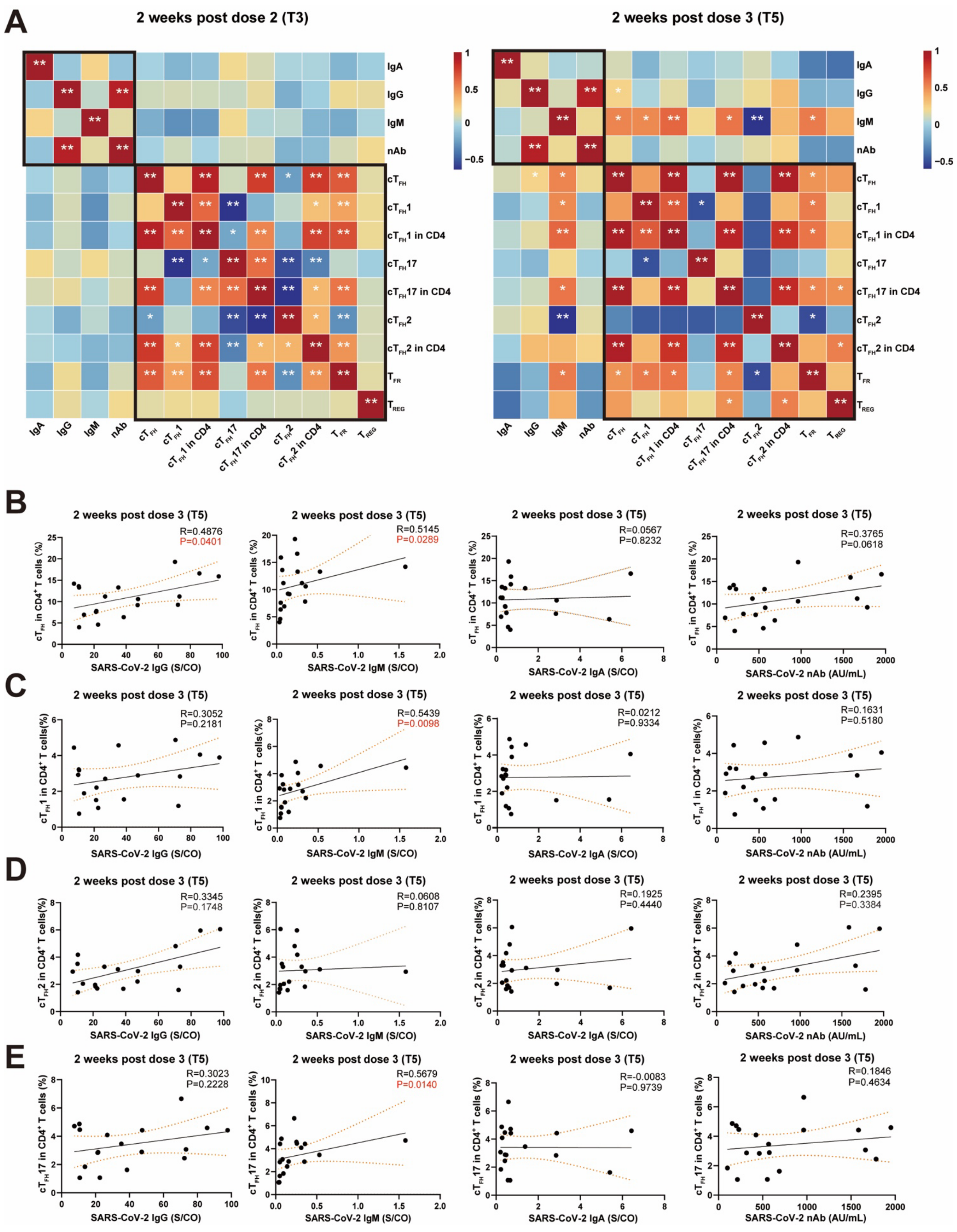

Supplementary figure S5: Correlations between polyclonal $CD4^+$ T cells and antibody responses following CoronaVac vaccination.

(A) Correlation heatmaps of the polyclonal $CD4^+$ T cell subsets and SARS-CoV-2 specific antibodies at two weeks post dose 2 (T3) and dose 3 (T5). Correlation analysis between the frequency of polyclonal cT_{FH} cells (B), $cT_{FH}1$ cells (C), $cT_{FH}2$ cells (D), $cT_{FH}17$ cells (E) and SARS-CoV-2-specific IgG, IgM, IgA and nAb titers at two weeks post dose 3 (T5). Each dot represents an individual subject. The non-parametric Spearman's rank correlation was used (A, B, C, D, E). *$P < 0.05$; **$P < 0.01$ (A). *P* and R values were indicated (B, C, D, E).

Supplementary table 1 Antibodies and other key resources

REAGENT or RESOURCE	SOURCE	IDENTIFIER
**Antibodies**		
Brilliant Violet 570 Anti-Human CD8a (Clone: RPA-T8)	BioLegend	Cat# 301038
Alexa Fluor 488 Anti-Human CD45RA (Clone: HI100)	BioLegend	Cat# 304114
Alexa Fluor 532 Hu CD3 (Clone: UCHT1)	eBioscience	Cat# 58-0038-42
PE Anti-Human CD25 (Clone: M-A251)	BioLegend	Cat# 356104
PE-Cy7 Anti-Human CCR7 (CD197) (Clone: 3D12)	BD Biosciences	Cat# 557648
APC Anti-Human CD185 (CXCR5) (Clone: J252D4)	BioLegend	Cat# 356908
APC-R700 Anti-Human CD127 (Clone: HIL-7R-M21)	BD Biosciences	Cat# 565185
APC/Fire 750 Anti-Human HLA-DR (Clone: L243)	BioLegend	Cat# 307658
Brilliant Violet 421 Anti-Human CD183 (CXCR3) (Clone: G025H7)	BioLegend	Cat# 353716
Super Bright 436 Anti-Human CD19 (Clone: HIB19)	eBioscience	Cat# 62-0199-42
BV480 Anti-Human Gma Dta TCR (Clone: B1)	BD Biosciences	Cat# 566076
Brilliant Violet 605 Anti-Human CD196 (CCR6) (Clone: G034E3)	BioLegend	Cat# 353420
Brilliant Violet 650 Anti-Human CD279 (PD-1) (Clone: EH12.2H7)	BioLegend	Cat# 329950
BV750 Anti-Human CD4 (Clone: SK3)	BD Biosciences	Cat# 566355
PE-Cy7 Anti-Human IFN-γ	BioLegend	Cat# 502528
APC-R700 Anti-Human IL-2	BD Biosciences	Cat# 565136

**Chemicals, Peptides, and Recombinant Proteins**		
7AAD Staining Solution	eBioscience	Cat# 00-6993-50
FACS Lysing Solution	BD Pharmingen	Cat# 349202
Human Fc Block	Miltenyi	Cat#130-059-901
Ficoll-Paque^{TM} PLUS Media	GE Healthcare Life Sciences	Cat#17144002
Cytofix/Cytoperm	BD Pharmingen	Cat#51-2090KZ
BD Perm/Wash	BD Pharmingen	Cat#51-2091KZ
FBS	Gibco	Cat#10100147
SARS-CoV-2 spike Peptide Pool	SinoBiological	Cat#PP003

SEB	Toxin Technology	Cat#BT-202
Purified anti-human CD3 Antibody	BioLegend	Cat#317326
Purified anti-human CD28 Antibody	BioLegend	Cat# 302933
Purified anti-human CD49d Antibody	BioLegend	Cat# 304339
**Critical Commercial Assays**		
Anti-SARS-CoV-2 IgG MCLIA kit	Autobio Diagnostics	Cat# CMU0302
Anti-SARS-CoV-2 IgM MCLIA kit	Autobio Diagnostics	Cat# CMU0402
Anti-SARS-CoV-2 IgA MCLIA kit	InnoDx Biotechnology	Cat# CT0725
SARS-CoV-2 neutralization antibody CLIA microparticles	Beckman Autobio Diagnostics	Cat# CMU0702
